# Perovskite single-crystal thin films: preparation, surface engineering, and application

**DOI:** 10.1186/s40580-023-00373-7

**Published:** 2023-05-22

**Authors:** Zemin Zhang, Wooyeon Kim, Min Jae Ko, Yuelong Li

**Affiliations:** 1 Institute of Photoelectronic Thin Film Devices and Technology, Key Laboratory of Photoelectronic Thin Film Devices and Technology of Tianjin, Engineering Research Center of Thin Film Optoelectronics Technology (MoE), Nankai University, Tianjin, 300350 China; 2 Department of Chemical Engineering, Hanyang University, 222 Wangsimni-ro, Seongdong-gu, Seoul, 04763 Korea

**Keywords:** Perovskite, Single crystal, Crystal growth, Surface engineering, Photovoltaic, Photodetector, Light-emitting device

## Abstract

Perovskite single-crystal thin films (SCTFs) have emerged as a significant research hotspot in the field of optoelectronic devices owing to their low defect state density, long carrier diffusion length, and high environmental stability. However, the large-area and high-throughput preparation of perovskite SCTFs is limited by significant challenges in terms of reducing surface defects and manufacturing high-performance devices. This review focuses on the advances in the development of perovskite SCTFs with a large area, controlled thickness, and high quality. First, we provide an in-depth analysis of the mechanism and key factors that affect the nucleation and crystallization process and then classify the methods of preparing perovskite SCTFs. Second, the research progress on surface engineering for perovskite SCTFs is introduced. Third, we summarize the applications of perovskite SCTFs in photovoltaics, photodetectors, light-emitting devices, artificial synapse and field-effect transistor. Finally, the development opportunities and challenges in commercializing perovskite SCTFs are discussed.

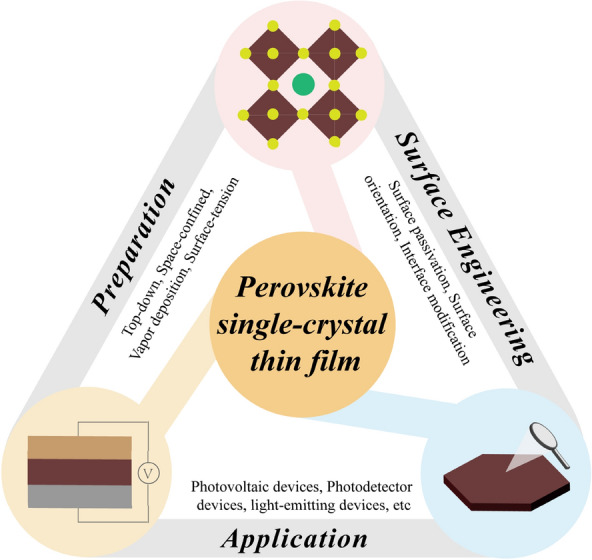

## Introduction

Halide perovskites (PVKs) possess suitable properties for thin-film optoelectronic devices, such as a high absorption coefficient and carrier diffusion rate, long carrier diffusion length and lifetime, and adjustable emission spectrum [1, 2]. Additionally, PVKs have the advantage of lower nucleation and crystallization activation energies compared with those of conventional semiconductor materials, allowing the preparation of films through low-temperature solution processes [3–6]. However, the conventional approach to fabricate PVK films results in the formation of polycrystalline films owing to the rapid volatilization of the solvent from a supersaturated precursor solution. These PVK polycrystalline films ultimately consist of small grains and have many grain boundaries, a mixed surface morphology, and a high density of surface and bulk defect states [7–9]. To address the issues associated with conventional polycrystalline PVK films, researchers have focused on the potential of single-crystal (SC) PVK.

Numerous experimental studies have verified that PVK SCs exhibit improved photoelectric properties compared to their polycrystalline counterparts [10–12]. Shi et al. demonstrated that MAPbI_3_ and MAPbBr_3_ SC materials have narrower bandgaps and wider absorption spectra than the corresponding polycrystalline films, which could enhance photon harvesting and hence improve photocurrent generation [13]. Moreover, PVK SCs exhibit a low defect density, resulting in a narrow and blue-shifted photoluminescence (PL) peak and high photoluminescence quantum yield (PLQY). Dong et al. also reported that the carrier diffusion length of the PVK SC is > 175 μm under 1 sun illumination and > 3 mm under weak illumination (0.003% sun), which results from the high carrier mobility, long carrier lifetime, and low trap densities [14]. Furthermore, the grain boundaries of PVK are highly susceptible to the penetration of external chemicals such as water and oxygen as well as the volatilization of internal components [15–17]. These grain boundaries also act as channels for ion migration, leading to current hysteresis [18, 19]. Consequently, PVK SCs without grain boundaries exhibit enhanced stability against external stimuli and negligible hysteresis.

A key point for advancing the development of optoelectronic devices is the preparation of PVK single-crystal thin films (SCTFs) with suitable thickness, large lateral dimensions, and high crystal quality. Recently, research on thickness control methods and optoelectronic applications of PVK SCTFs has received significant attention. The practical application of these films was realized by precisely controlling the concentration of the precursor solution, growth temperature, and thickness of the active layer [20, 21]. Although thickness-controllable PVK SCTFs grown in-situ on the underlayer have been successfully prepared, the performance of PVK SCTFs devices is not as high as that of polycrystalline film devices. The device performance is mainly limited by the surface defects of the PVK SCTFs [22]. A drive-level capacitance profiling (DLCP) test showed that the trap density decreases from the interface to the interior of the SC owing to the formation of charge traps by the suspended bonds on the surface [23]. The surface recombination velocity (SRV) of an SC sample is more than six times higher than that of a polycrystalline sample, which reduces the carrier lifetime and decreases the device performance in the case of the former [24]. Calculations showed that reducing the surface defect density is favorable for developing high-efficiency SC-PVK-based devices. The number of surface defects generated is related to the conditions used to prepare the PVK SCTFs [25]. Different preparation conditions result in different surface termination atoms, which determine the surface photoelectric characteristics. For example, the loss of methylammonium iodide (MAI) under high-temperature heating conditions results in a PbI_2_-rich surface that traps carriers. Furthermore, the strain caused by the significant lattice mismatch between the substrate and subsequent layers generates additional defects [26–29]. Overall, it is necessary to conduct in-depth research on optimizing the preparation conditions and reducing the surface defects of PVK SCTFs to promote the commercial application of high-performance and high-stability PVK SCTF optoelectronic devices.

Here, we summarize the nucleation, growth, and methods of preparing PVK SCTFs. The preparation methods are classified based on the differences in the growth conditions, such as top-down, space-confined, surface tension, and vapor deposition methods. We focus on surface engineering to reduce the surface defect density and optimize the interfacial properties of PVK SCTF devices. This report focuses on three important aspects: surface passivation, interface modification, and surface orientation engineering. Furthermore, we provide examples of the application of PVK SCTFs in photovoltaics, photodetectors, light-emitting devices, and artificial synaptic devices. These examples demonstrate that PVK SCTFs have significant application potential in the field of optoelectronic devices. This review provides guidance for research on the preparation and application of PVK SCTFs.

## Preparation of PVK SCTFs

Recently, various strategies based on bulk SCs have been developed for preparing PVK SCTFs. Different preparation methods significantly affect the photoelectric properties of PVK SCTFs. The understanding of the crystallization process and optimization of the preparation methods have enabled the preparation of high-quality PVK SCs with controllable thickness for PVK SCTFs devices.

### Nucleation and growth of SCTFs

**Fig. 1:**
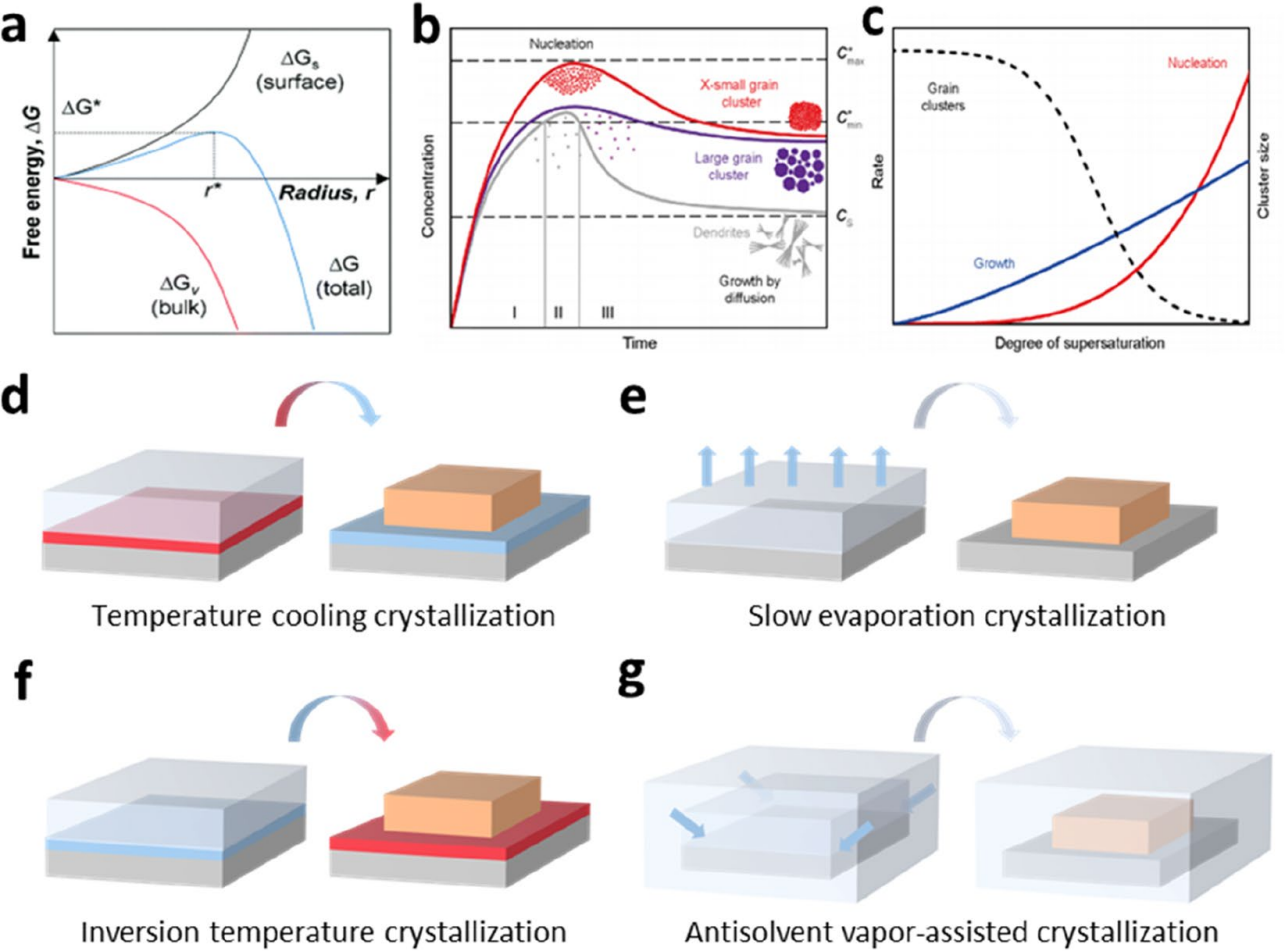
**a** Schematic of the classical free energy diagram for homogeneous nucleation as a function of the particle radius. Reproduced with permission from Ref. [33]. Copyright 2019, The Royal Society of Chemistry. **b** LaMer model for nucleation and crystal growth. Regions I, II, and III represent prenucleation, nucleation, and growth stages, respectively. **c** Nucleation (red trace) and growth (blue trace) rates as a function of the supersaturation degree. Reproduced with permission from Ref. [34]. Copyright 2017, Springer nature. Preparation methods of PVK SCs are categorized as **d** temperature cooling crystallization, **e** slow evaporation crystallization, **f** inversion temperature crystallization, and **g** antisolvent vapor-assisted crystallization

This section explains the classical nucleation and growth mechanisms before introducing the PVK SCTF preparation method. The crystallization process of PVK begins with the nucleation of the precursor complex in a supersaturated solution, followed by the formation of crystals through a self-assembly growth process [30–32]. Nucleation occurs when the concentration of the growing monomers is sufficiently higher than the solubility limit, leading to supersaturation. As shown in Fig. 1a, the total free energy (*ΔG*) is the sum of the surface free energy (*ΔG*_*s*_), that is, the free energy between the particle surface and the bulk of the particle, and the bulk free energy (*ΔG*_*v*_), that is, the free energy between a substantially large particle and the solute in solution [33]. With increasing radius (*r*) of a spherical particle in solution, *ΔG* first increases and then decreases. The thermodynamical critical radius (r*) of the nucleus determines the nucleation process in solution, ensuring that the nucleus can grow further and not dissolve again. When the radii of the nuclei are smaller than r*, the nuclei dissolve back into the solution. In contrast, nuclei with radii over r* can cause nucleation. Therefore, to preferentially crystallize the SC-PVK, it is necessary to control the conditions to form nuclei with radii larger than r*.

The LaMer model (Fig. 1b) was introduced to illustrate the processes of nucleation and subsequent growth. In this model, the crystallization process is divided into three stages [34]. In the first stage (pre-nucleation stage), the concentration of the solute surpasses the solubility limit (*C*_*s*_) and approaches supersaturation. In the second stage (nucleation stage), the concentration exceeds the critical level (*C*_min*_) for nucleation, and nucleation is significantly accelerated when the monomer concentration reaches a maximum level (*C*_max*_). At this stage, the accumulation and consumption of solutes are balanced by both nucleation and growth. The competition between nucleation and growth kinetics happened in this stage is a function of the solution supersaturation (Fig. 1c). In the third stage (growth stage), when the concentration of the growth species is below *C*_min*_, nucleation stops, and only crystal growth occurs. Additionally, smaller particles redissolve and redeposit on larger crystals, which is a phenomenon known as Ostwald ripening. Therefore, to obtain high-quality PVK SCs, a strategy to suppress the nucleation process and enhance the growth process is essential. Lian et al. selected a small seed crystal as a single nucleation site for the growth of PVK SCs [35]. Ma et al. demonstrated that the nucleation rate can be reduced by introducing a polymer coordinated with Pb^2+^ to inhibit the nucleation of impurity crystals [36]. Chen et al. introduced surfactants to control the nucleation density and promote the anisotropic growth of high-quality PVK SCs [37]. Moreover, for sustained crystal growth, it is imperative to maintain conditions that sustain the growth stage. Solution dynamic-flow reaction systems are widely used to continuously provide solutions for crystal growth [38, 39].

Based on the nucleation and crystal growth mechanisms, the proposed methods for preparing PVK SCs can be classified as follows: (1) temperature cooling crystallization methods (Fig. 1d): These methods are generally used to synthesize SC materials and are simple to execute; however, they have a long growth time [35]. The related principle is that by lowering the temperature of the saturated precursor solution, the crystals slowly precipitate out of the solution as the solubility of the solute decreases. (2) Slow evaporation crystallization methods (Fig. 1e): The crystallization of PVK crystals occurs during the slow evaporation of the solvent in a saturated solution [15, 40, 41]. Generally, the evaporation of the solvent is accelerated by heating; however, the nucleation process is difficult to control. (3) Inversion temperature crystallization methods (Fig. 1f): Temperature cooling and slow evaporation crystallization methods generally require a long synthesis time and have a low yield, which are not conducive to the production and practical application of PVK SCs. Inversion temperature crystallization benefits from the unique temperature-dependent properties of PVK and is the commonly used preparation method at present [42–44]. (4) Antisolvent vapor-assisted method (Fig. 1g): The principle involves the diffusion of an antisolvent of the solute into a saturated solution of the precursor, which significantly reduces the solubility of the solute in the system, followed by precipitation crystallization [13]. The preparation of PVK SCTFs with controllable thickness is based on preparation methods using other physical or chemical pathways to inhibit the growth in the longitudinal direction of SCs and promote growth in the lateral dimension.

### Top-down method

**Fig. 2:**
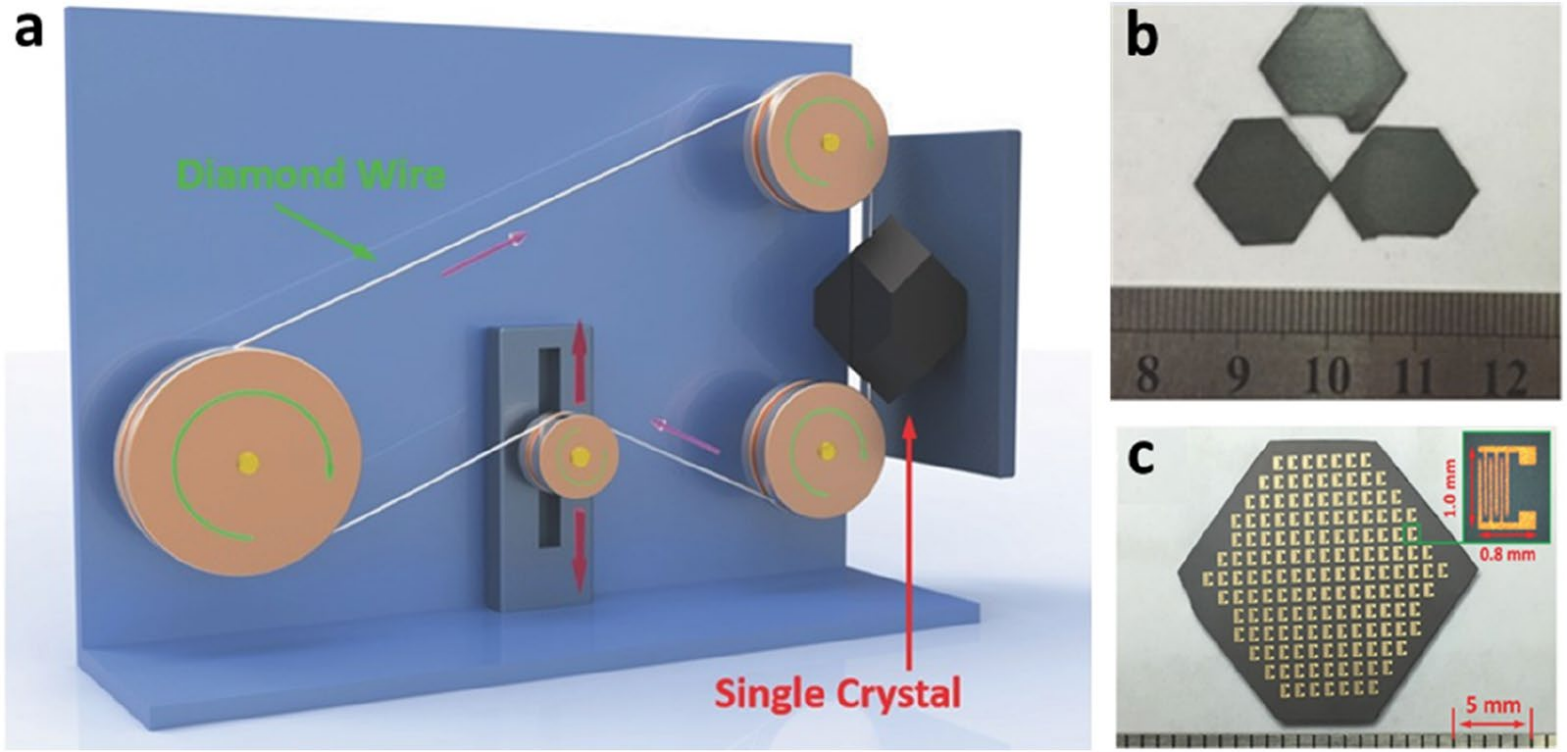
Top-down method for fabricating PVK wafers. **a** Slicing process for fabricating FAPbI_3_ SC wafer. **b** Top view of FAPbI_3_ SC wafers. **c** Pictorial representation of an array of 153 integrated photodetectors fabricated on a PVK wafer. Reproduced with permission from Ref. [45]. Copyright 2016, WILEY-VCH

Large-sized wafers are the foundation materials for fabricating advanced integrated semiconductor devices. However, preparing industrial PVK wafers using the top-down method remains challenging. In 2016, Liu et al. first prepared large PVK SC wafers using seed crystals and inversion crystallization strategies [45]. The crystal was sliced into wafers using diamond wires (Fig. 2a). The key experimental parameters are the linear sawing speed and crystal feeding speed of the slicing machine. After optimizing the process, a PVK SC wafer with a definite shape and thickness of 100 μm was successfully prepared (Fig. 2b). One-hundred-and-fifty-three photodetector arrays were designed and fabricated on a large scale on a wafer, enabling a higher light response and a wider light absorption range than those achieved with the corresponding thin-film devices (Fig. 2c). The aforementioned study proves the feasibility of manufacturing integrated circuits on large PVK wafers. Based on their previous research, Liu et al. prepared a PVK SC wafer with a large size and expanded the experiment to double halide PVK crystal materials [46]. The difficulty in slicing large crystals into thin wafers is that the crystals can easily crack or burn, resulting in surface defects or incomplete surfaces. Adapting PVK materials to the water-cooling process of silicon wafer production lines is difficult because of their water sensitivity. Therefore, a MAI-saturated iso-propylalcohol (IPA) solution was developed as the coolant. The resulting PVK SC wafer has a low defect state density and high temperature stability. However, owing to the complexity of the experiment and operation as well as the lack of manufacturing equipment and experience in this field, few researchers choose to use the top-down method to prepare PVK SCTFs. Further advancements in this field require the development of a suitable route for PVK wafer manufacturing.

### Space-confined growth method

**Fig. 3:**
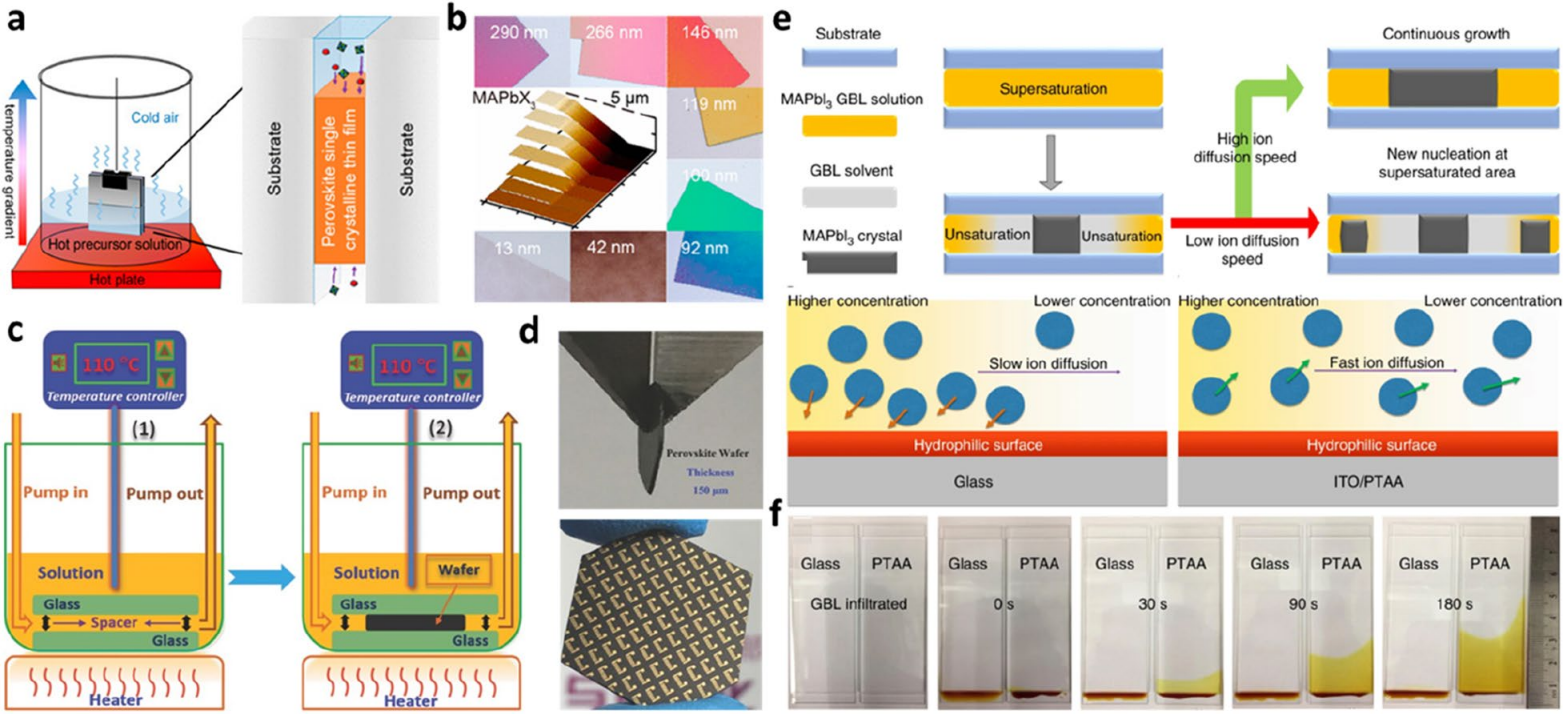
Space-confined growth of PVK SCTFs. **a** Facile space-confined solution-processing strategy for growing PVK SCTFs. **b** Optical images of ultrathin MAPbBr_3_ SCTFs showing the thickness-dependent colors. Reproduced with permission from Ref. [47]. Copyright 2016, American Chemical Society. **c** Fabrication of ultrathin PVK SCTFs using a dynamic flow microreactor system. **d** Optical images of ≈ 150 μm-thick wafer and integrated photodetectors fabricated on the PVK SC wafer. Reproduced with permission from Ref. [48]. Copyright 2016, WILEY-VCH. **e** Schematic of ion diffusion processes in PVK SCTFs using hydrophilic and hydrophobic substrates. **f** Photographs of the diffusion of the MAPbI_3_ precursor solution on hydrophilic glass and hydrophobic PTAA-covered indium tin oxide (ITO) substrates after different times. Reproduced with permission from Ref. [49]. Copyright 2017, Springer Nature

In the preparation of halide PVK materials, the low-temperature solution process provides opportunities for low-cost and low-power industrialization. Chen et al. reported a facile space-confined growth strategy for preparing PVK SCTFs via a solution process, the main principle of which is that a growth space is formed in the middle of two substrates (Fig. 3a) [47]. The precursor solution is injected between the two substrates using a capillary force. The convection of the solution caused by the temperature gradient from the bottom to the top provides the growth materials. As shown in Fig. 3b, the thickness of the SC film can be controlled by confining the space between the substrates. Finally, MAPbBr_3_ SCTFs of different thicknesses without significant defects were obtained, and they exhibited bright and uniform luminescence. Liu et al. reported a dynamic flow microreactor system for the large-scale production of PVK SCTFs with a defect state density of ~ 6 × 10^8^ cm^− 3^ (Fig. 3c) [48]. The dynamic material replenishing process induced continuous crystal growth, resulting in high-quality SC wafers, which were used to integrate 100 photodetectors (Fig. 3d). Huang’s group further investigated the influence of hydrophilic and hydrophobic substrates on the space-confined growth of SCTFs (Fig. 3e) and fabricated MAPbI_3_ SCTF solar cells [49]. The photographs in Fig. 3f show that the precursor solution between two hydrophobic poly[bis (4-phenyl) (2,4,6-trimethylphenyl)amine] (PTAA) substrates has a high diffusion speed and long diffusion distance. The low surface tension of the hydrophobic substrate significantly increased the ionic diffusion rate of the solution, leading to the continuous growth of the SC film in the plane direction. They found that the bulk defects of MAPbI_3_ SCTFs were reduced; however, carrier recombination on the surface of the SCTFs increased. This may be because MAI molecules were lost from the surface of the MAPbI_3_ SCTFs when the crystals were removed from the hot solvent. The power conversion efficiency (PCE) of the optimal MAPbI_3_ SCTF solar cell was 17.8% after the surface passivation process. Furthermore, researchers have studied photodetector devices based on PVK SCTFs. Yang et al. optimized the inversion temperature crystallization method and the space-confined growth method for fabricating MAPbBr_3_ SCTFs [50]. The thickness of the SCTF, which reached the order of 100 nm, was precisely adjusted by controlling the pressure of the upper substrate. The surface modification of the substrate was performed to regulate nucleation and crystal growth to obtain high-quality SCTFs and prepare high-performance photodetectors.

**Fig. 4:**
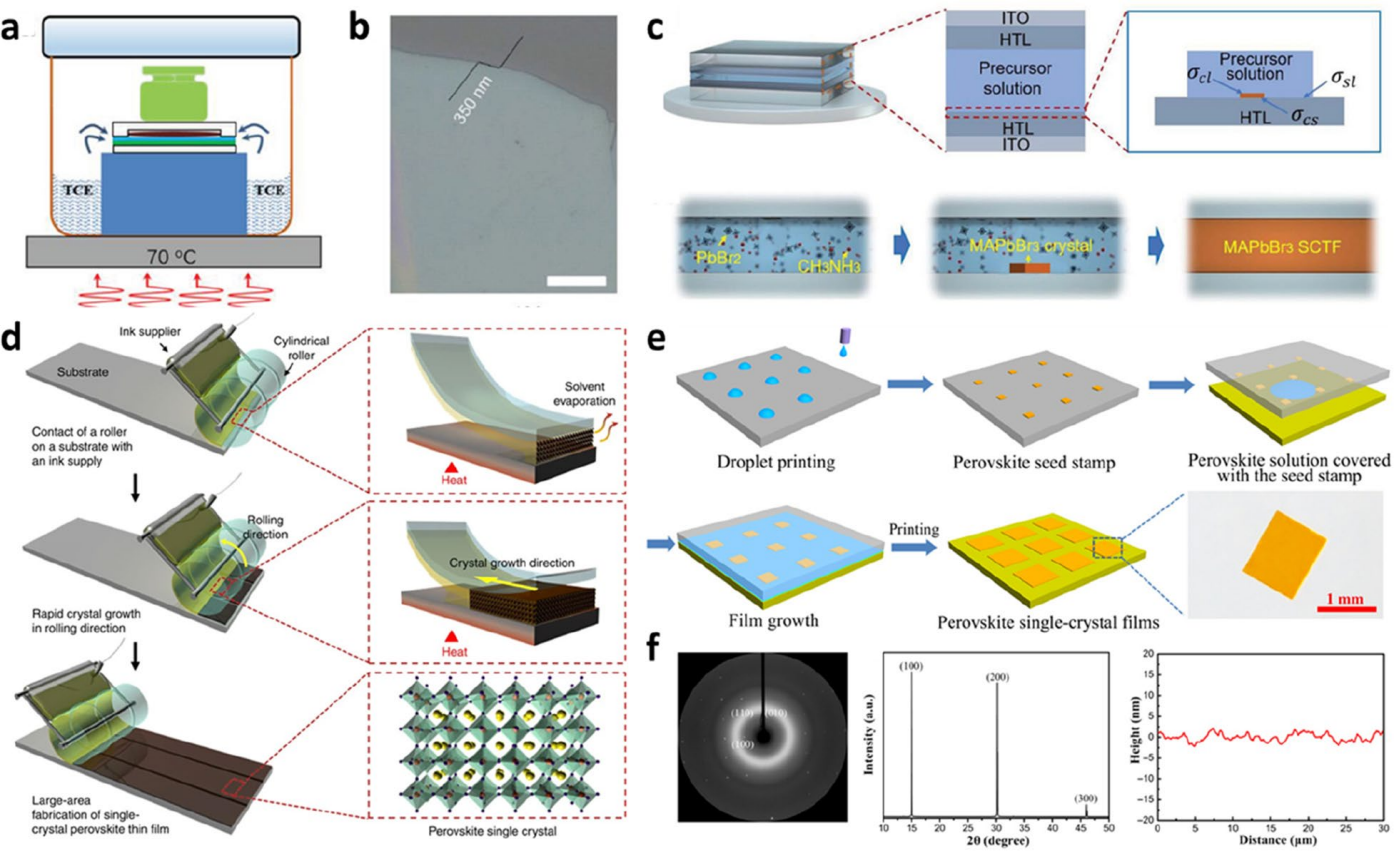
Space-confined growth of PVK SCTFs. **a** Antisolvent-assisted crystallization and space-confined strategies for growing MAPbI_3_ SCTFs. **b** Optical image of MAPbI_3_ SCTF (scale bar is 50 μm). Reproduced with permission from Ref. [51]. Copyright 2020, WILEY-VCH. **c** Schematic of the nucleation and growth of MAPbBr_3_ SCTF from precursor solution in a confined space. Reproduced with permission from Ref. [52]. Copyright 2022, WILEY-VCH. **d** Schematic of geometrically confined lateral crystal growth of large-area PVK SCTFs using the roller coating method. Reproduced with permission from Ref. [54]. Copyright 2017, Springer Nature. **e** Seed printing process for the scalable growth of PVK SCTFs. **f** X-ray diffraction (XRD) patterns and atomic force microscopy (AFM) profile of PVK SCTFs. Reproduced with permission from Ref. [55]. Copyright 2018, AAAS

Recently, new preparation methods based on the confined-space method have been developed. Kong et al. combined an antisolvent-assisted crystallization method with the space-confined method (Fig. 4a) [51]. Trichloroethane was used as the antisolvent to prepare high-quality millimeter-level MAPbI_3_ PVKs at 70 °C. The thickness of the SCTFs was controlled from tens of nanometers to several micrometers (Fig. 4b). Tang et al. conducted an in-depth study on the growth of high-quality SCTFs and found that the growth was affected by three key factors: the interfacial energy between the precursor solution and substrate, heating rate, and precursor solution concentration (Fig. 4c) [52]. Under the optimized conditions, large SCTFs with high quality and long-term stability were grown in situ on the poly(*N,N*″-bis-4-butylphenyl-*N,N*″-bisphenyl)-benzidine (poly-TPD) hole transport layer (HTL). Li et al. proposed a space-confined growth model for ultrathin SCTFs in interfacial electric fields, driven by solid–liquid phase charge separation [53]. The cracked mica substrate had a high surface charge and formed an interfacial electric field, which was conducive to controlling the distribution of the precursor. High local saturation resulted in the preferential nucleation and growth of the crystals along the lateral dimension. This method can also be used to prepare different types of ultrathin PVK materials.

The large-scale preparation of PVK SCTFs via the solution method has also attracted considerable attention. Lee et al. reported a roll-to-roll printing method for the large-scale manufacture of PVK SCTFs [54]. As shown in Fig. 4d, the prepared PVK precursor ink was transferred to a hot substrate through a rolling mold with a pattern. Upon the evaporation of the solvent, the PVK SCTFs underwent continuous space-confined crystallization. Gu et al. demonstrated a large-scale PVK SCTF printing method (Fig. 4e) [55]. The principle involves preparing the seed crystal template via the inkjet printing method, followed by its transfer to the substrate with the precursor solution. From the results shown in Fig. 4f, the growth of the seed crystals enabled the formation of high-quality PVK SCTFs with a smooth morphology. The advantage of this method is that the nucleation location can be selected via the printing technology to achieve an ultralow nucleation density.

### Surface tension-assisted growth method

**Fig. 5:**
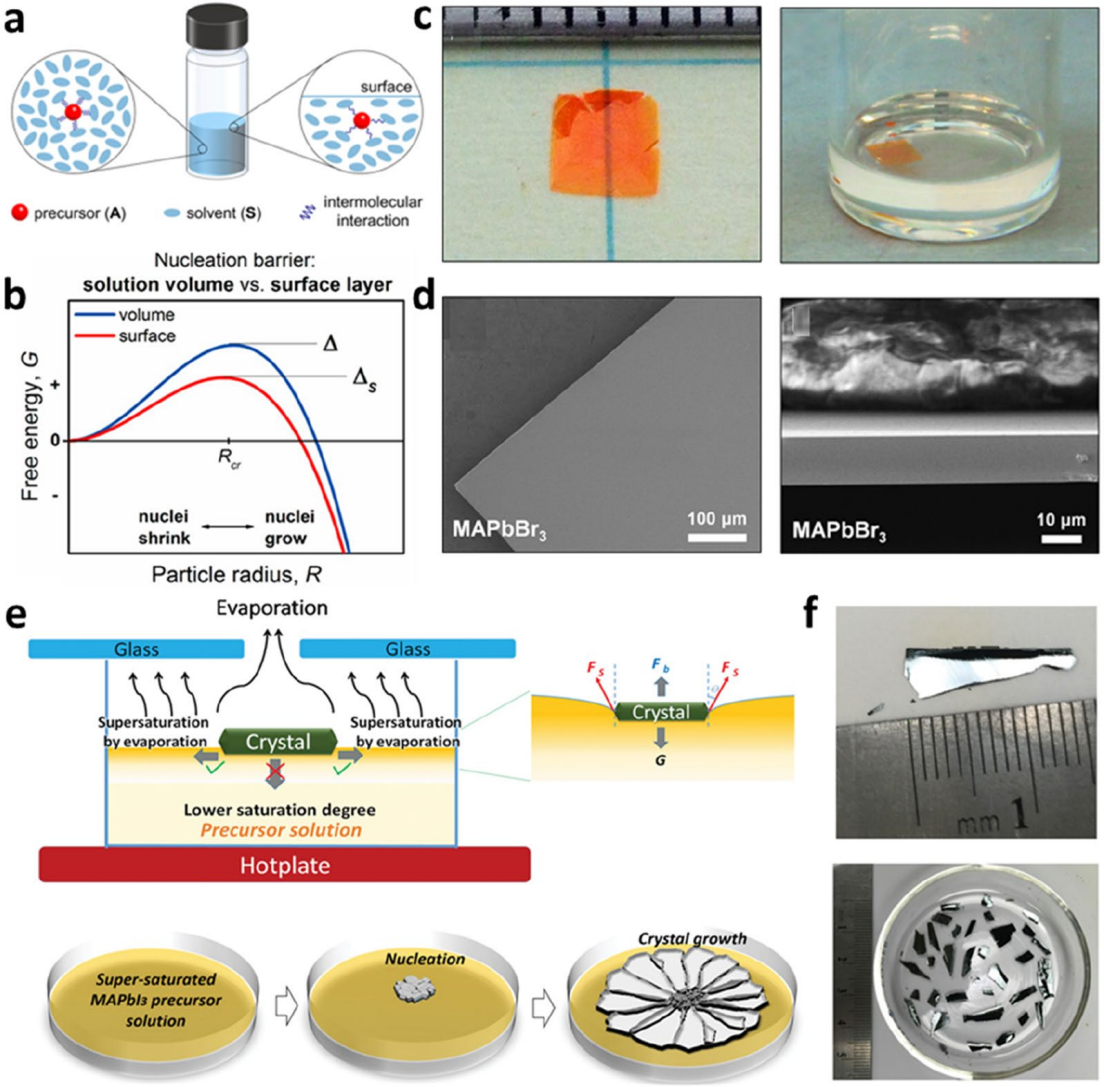
Surface tension-assisted growth of PVK SCTFs. **a** Schematic of molecular interaction in the volume (left) and the surface layer (right) of the solution. **b** Graph of relationship between the free energy and particle radius, revealing a lower nucleation barrier of the solution surface layer (red line) than that in the solution volume (blue line). **c** Photographs of the MAPbBr_3_ SCTF. **d** Top-view and side-view SEM images of the MAPbBr_3_ SCTF. Reproduced with permission from Ref. [56]. Copyright 2017, American Chemical Society. **e** Scheme of the nucleation and crystal growth of the MAPbI_3_ SCTF driven by the evaporation of the solution. **f** Photographs of MAPbI_3_ SCTFs. Reproduced with permission from Ref. [57]. Copyright 2019, WILEY-VCH

The preparation of PVK SCTFs with high quality and a large aspect ratio is important for broadening their applications. Bakr et al. revealed the role of the surface tension of the solution in the preparation of PVK SCTFs [56]. As shown in Fig. 5a, increasing the distance between molecules in the surface layer reduces the interaction energy, resulting in a low nucleation barrier in the surface layer (Fig. 5b). During the growth process, the precursor molecules are adsorbed on both sides, and the aspect ratio of the SC film increases gradually. The photographs (Fig. 5c) and scanning electron microscopy (SEM) images (Fig. 5d) show that PVK SCTFs with a higher aspect ratio and good morphology were successfully prepared. However, the trap state density of the PVK SCTF was slightly higher than those of the bulk SCs because surface traps were induced by the large contact area with the solution. Their study has considerable significance for the preparation of PVK SCTFs with a high aspect ratio and paves the way for achieving PVK crystallization controlled by the surface tension at the air–solution interface. Liu et al. improved the surface tension-assisted growth of PVK SCTFs (Fig. 5e) by regulating the evaporation of the solution by controlling the gap between the two glasses on the beaker [57]. It was also found that the crystal thickness can be controlled by the gap distance, solution height, and heating temperature. Photoelectric characterization proved that the as-prepared PVK SCTF had a long charge recombination lifetime and broad absorption spectrum. A PVK SCTF solar cell with a lateral structure was designed, achieving a PCE of 5.9%. Wang et al. synthesized quasi-two-dimensional (2D) PVK SCTFs using a surface tension-assisted method [58]. The alkyl ammonium cations with hydrophilic heads were orderly arranged at the interface through static coulombic interactions, achieving uniform orientation and rapid in-plane growth. Photodetectors based on the quantum-well thickness (*n* = 1) of the quasi-2D PVK SCTFs exhibited a low dark current, high switching ratio, and short response time. Their study demonstrated the strong potential of emerging quasi-2D PVK materials in the field of SC photoelectric devices.

### Vapor deposition growth method

**Fig. 6:**
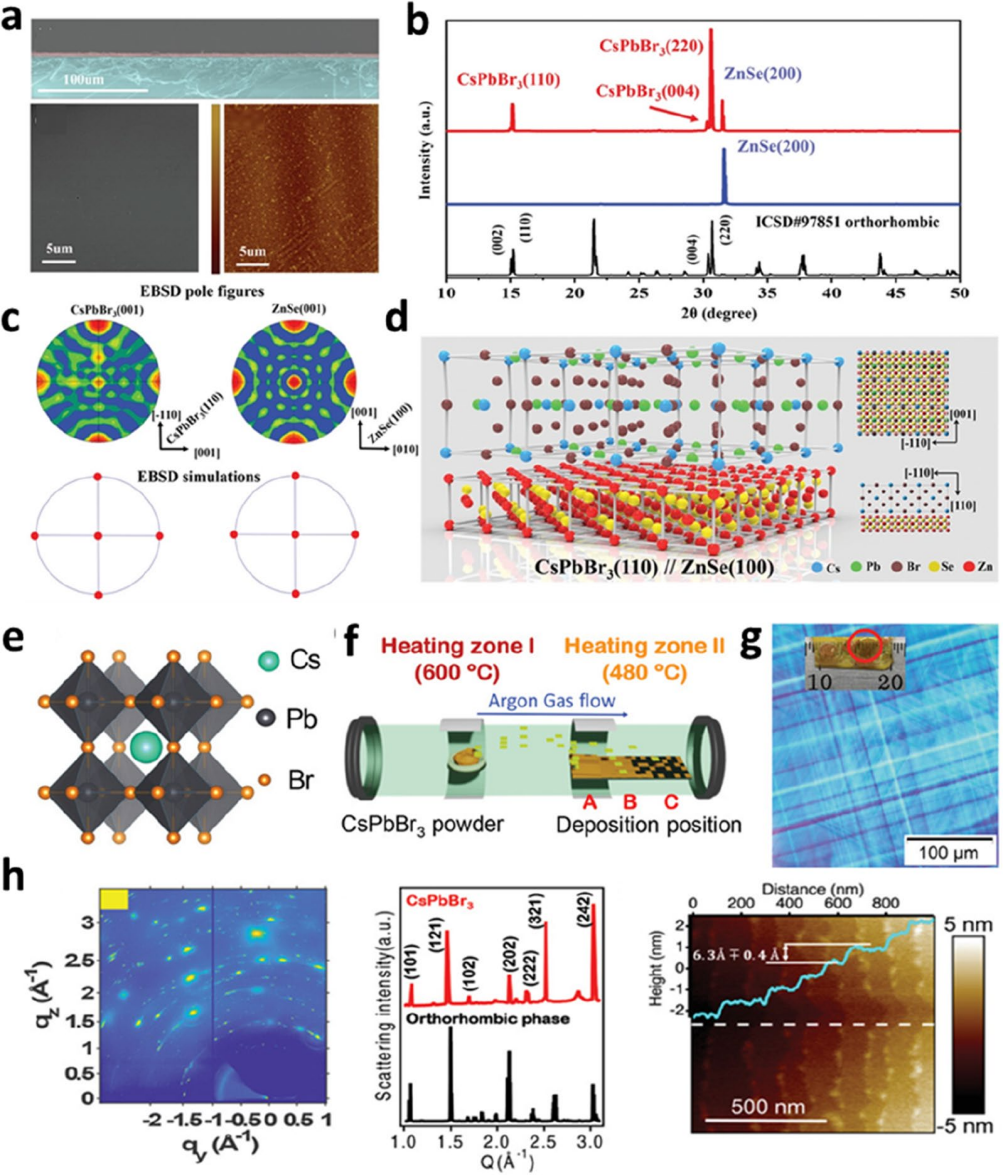
Growth of PVK SCTFs via vapor deposition. **a** Characterization of the surface morphology of the CsPbBr_3_ SCTF, which was epitaxially grown on a ZnSe (100) substrate. **b** XRD patterns of the CsPbBr_3_ SCTF (red line), ZnSe (100) (blue line), and reference (black line). **c** EBSD pole figures and simulations for CsPbBr_3_ (left) and ZnSe (right). **d** Schematic of the epitaxial growth of the CsPbBr_3_ SCTF on the ZnSe (100) substrate. Reproduced with permission from Ref. [61]. Copyright 2019, WILEY-VCH. **e** Crystal structure of CsPbBr_3_. **f** Schematic of the fabrication of the CsPbBr_3_ SCTF via the CVD method. **g** Polarized optical microscopy image of a single grain. Inset shows the photograph of the CsPbBr_3_ SCTF with mm-scale grains. **h** Crystal and surface characterizations of the CsPbBr_3_ SCTF. Reproduced with permission from Ref. [62]. Copyright 2021, WILEY-VCH

The vapor deposition method for growing PVK SCTFs mainly comprises the deposition of the precursor on the substrate and the subsequent epitaxial crystal growth. To select a suitable method for epitaxial film growth, the thermodynamic and kinetic conditions for the growth of the crystalline material as well as the lattice matching, surface energy, and thermal expansion coefficient of the substrates and materials should be considered. Chen et al. used a PVK oxide (SrTiO_3_; STO) as the substrate for the epitaxial growth of a CsPbBr_3_ nanoplate array and obtained an SC film with a controllable thickness [59]. Although the sizes of these two lattices are different, the two CsPbBr_3_ units match the three STO units, resulting in a final lattice mismatch factor of only 0.47%. Therefore, CsPbBr_3_ SCTFs achieved good heteroepitaxial growth. Increasing the reaction temperature mitigated the probability of the Volmer–Weber (V–W) mode growth in the CsPbBr_3_ SCTFs because high temperatures can enhance the diffusion of adsorbed atoms and accelerate the nucleation of epitaxial crystals. This study provides guidance for fabricating high-performance devices with epitaxially grown PVK SCTFs. Wang et al. realized the high-temperature gas-phase epitaxial growth of PVK SCTFs on a NaCl substrate [60]. NaCl materials have the same cubic symmetry and lattice and chemical properties as halide PVK materials, rendering them suitable as heterogeneous epitaxial growth substrates. The experimental results showed that the prepared PVK SCTF had centimeter-scale lateral dimensions, nanometer to micrometer thickness, and excellent carrier dynamic characteristics. Meng’s group prepared orthorhombic CsPbBr_3_ SCTFs via vapor-phase epitaxial growth on a ZnSe substrate [61]. The morphological characterization data in Fig. 6a show that the CsPbBr_3_ SCTF has a smooth surface and a clear crystal interface. The XRD patterns (Fig. 6b) and electron backscatter diffraction (EBSD) pole figures (Fig. 6c) demonstrate that the CsPbBr_3_ SCTFs have a high crystal quality, and their crystal structure and orientation match those of ZnSe. A three-dimensional (3D) diagram of the heteroepitaxial growth of CsPbBr_3_ (110) on the ZnSe (100) substrate is shown in Fig. 6d. Zhou et al. prepared high-quality and large-sized all-inorganic PVK SCTFs via the chemical vapor deposition (CVD) of presynthesized CsPbBr_3_ microcrystalline powder (Fig. 6f) [62]. By optimizing the flow rate, deposition location, and temperature, the lateral dimension of the CsPbBr_3_ SCTFs was increased to the millimeter scale. Crystal and surface characterizations proved that the obtained CsPbBr_3_ film had a high crystal quality (Fig. 6h). Moreover, a lateral-structure diode was prepared using large grains. A high light-generation voltage and photocurrent at zero bias were observed under photoillumination, which is indicative of efficient carrier transport and collection in the long channel in the crystal.

## Surface engineering of PVK SCTFs

Although the bulk defects of PVK SCs are significantly low owing to the periodically ordered lattice, the exsist of surface defects of PVK SCs cannot be ignored [63–65]. Ni et al. used the drive-level capacitance profiling (DLCP) method to analyse spatial distributions of carrier and trap densities in perovskites [23]. The trap density varied by five orders of magnitude from surface to film interior, and most of the deep traps located at crystal surfaces. The different structure, morphology, and distribution of electronic states on the surface of PVK SCs affect the photoelectric properties of the surface such as carrier dynamics, photocurrent, and optical band gap. In PVK materials, the common defects include vacancy, interstitial, and antisite defects, which will produce a defect state energy level [66–68]. Suspension bonds, dislocations, and chemical contaminants exist on the surface of PVK SCs [69]. These defects on the surface of PVK SCs may be derived from crystal growth and processing. The possible sources of surface defects are the surface damage caused by cutting in the manufacture of PVK SCs wafer, the surface stress caused by the mismatched lattice constant and thermal expansion coefficient between the crystal and the cover plate or substrate, and the hydration in the environment [70]. PVK SCTFs surface defects trap charge carriers and promote recombination, which slows down carrier transport. They may also cause ion migration problems, which seriously affect the performance of PVK SCTFs devices. In the studies of polycrystalline PVK materials, it has been proved that surface engineering methods were helpful to deal with the problem of surface defects. Recently, researchers are trying to reduce the defects of SCTFs surface through a series of surface engineering techniques, such as using optimized preparation methods, adding surface passivation agents, and conducting hydrophobic post-treatment methods [71]. The above contents will be summarized and explained in detail in the following sections.

### Surface passivation

**Fig. 7:**
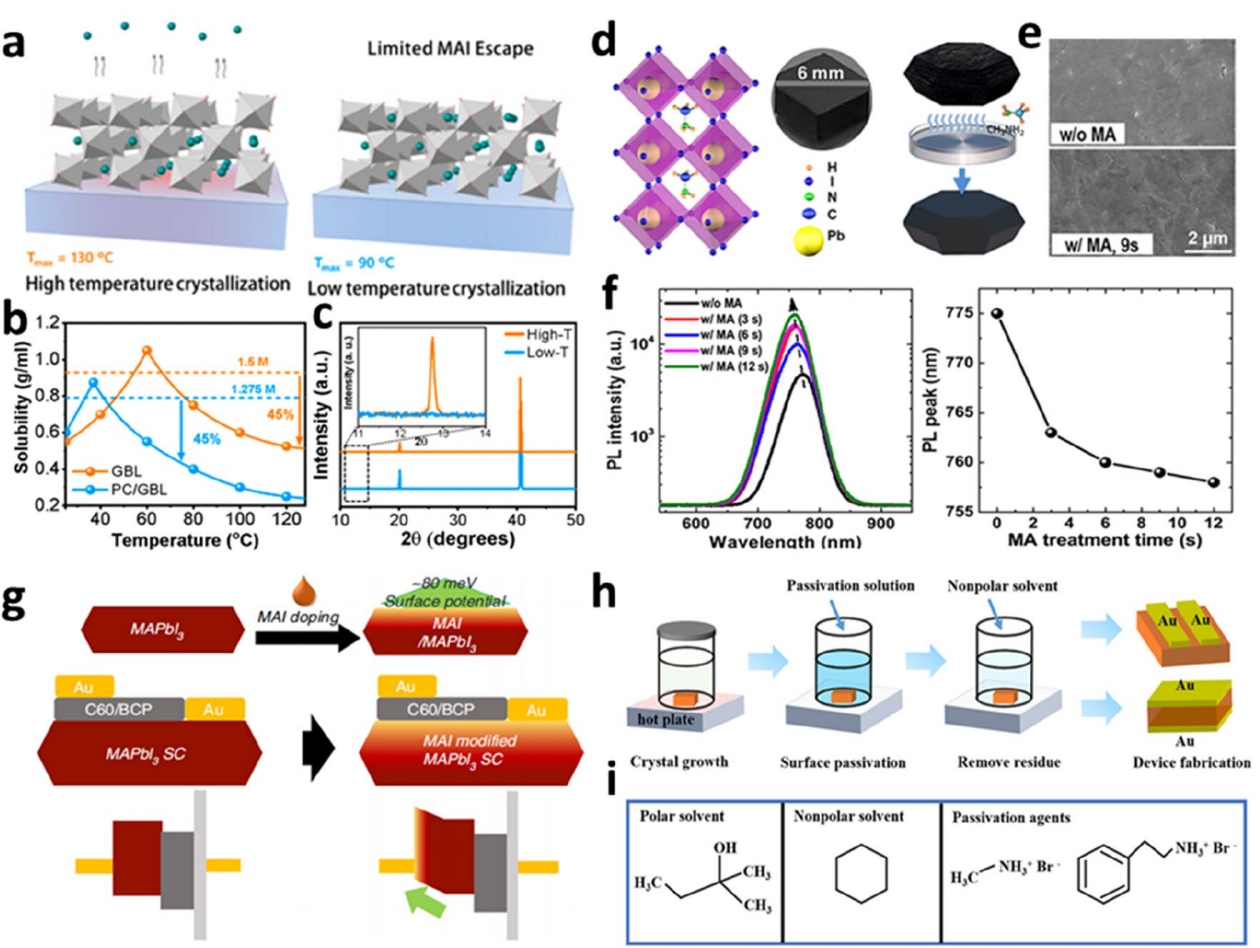
Surface passivation engineering of the PVK SC. **a** Schematic of the crystallization of the MAPbI_3_ SCTF at high (left) and low (right) temperatures. **b** Temperature-dependent solubility of MAPbI_3_ in GBL (orange line) and PC/GBL (blue line) solvent mixture. **c** XRD patterns of the MAPbI_3_ SCTF grown at high (orange line) and low (blue line) temperatures. Reproduced with permission from Ref. [72]. Copyright 2020, American Chemical Society. **d** Crystal structure of the MAPbI_3_ SC, and schematic of the surface treatment of the SC using MA gas. **e** SEM images of the MAPbI_3_ SC without and with MA gas surface treatment. **f** PL spectra of the MAPbI_3_ SC without and with MA gas surface treatment. Reproduced with permission from Ref. [73]. Copyright 2018, American Chemical Society. **g** Schematic of the MAI surface treatment on the MAPbI_3_ SCTF. Device structures and energy levels for the MAPbI_3_ SCTF solar cells without and with MAI surface treatment. Reproduced with permission from Ref. [74]. Copyright 2020, Springer Nature. **h** Schematic of the surface treatment and device fabrication of the MAPbBr_3_ SC. **i** Polar solvent is *tert*-amyl alcohol; the nonpolar solvent is cyclohexane, and the passivation agents are methylammonium bromide and phenylethylammonium bromide. Reproduced with permission from Ref. [75]. Copyright 2022, American Chemical Society

A strategy for reducing the high defect density of the SCTF surface is to precisely control the temperature conditions of the SC growth process. During the growth of the PVK SCTFs via the inversion temperature method, high temperatures above 120 °C cause the MAI to escape, forming many defects on the surface of the single crystal. Alsalloum et al. used a solvent engineering method to prepare SCTFs with a mixture of propylene carbonate (PC) and gamma-butyrolactone (GBL) (Fig. 7a) [72]. The mixed solution enabled the growth of MAPbI_3_ SCTFs at temperatures below 90 °C (Fig. 7b). As shown in Fig. 7c, the quality of the MAPbI_3_ SCTF grown at low temperatures was improved. The optimal MAPbI_3_ SCTF solar cell exhibited a high open-circuit voltage (*V*_OC_) of 1.15 V and a PCE of 21.9%. In addition to controlling the crystal growth conditions, passivating the defects on the surface using additives through a post-treatment process is another effective strategy. Zhou et al. studied the regulation of the photophysical properties, surface defects, and recombination of MAPbI_3_ SCTFs by methylamine (MA) steam surface treatment (Fig. 7d) [73]. Steady-state PL spectra (Fig. 7f) showed that the PL peak intensity was enhanced after the MA evaporation treatment. The low degree of nonradiative recombination was attributed to the Lewis acid–base passivation effect of the lead ions and MA. The suppression of surface defects contributed to carrier transport and collection. Song et al. fabricated a lateral-structured PVK SCTF solar cell with optimized anode contact through the MAI treatment of the SCTF surface [74]. Introducing an ultrathin MAI layer shifted the surface potential of the PVK SC toward that of the valence band (Fig. 7g). The improved energy level matching significantly enhanced the *V*_OC_ and fill factor (FF) of the device. Surface passivation resulted in a high conductivity of the MAPbI_3_ SCTF surface, demonstrating efficient charge collection. Chen et al. also demonstrated a solvent-process surface treatment for passivating halogen vacancy defects in MAPbBr_3_ SCs (Fig. 7h) [75]. They immersed the prepared SCs in a solution containing the halide passivators MABr and PEABr and washed them with a nonpolar solvent (molecular formulas are shown in Fig. 7i). The passivated MAPbBr_3_ SC showed a strong PL intensity and long PL lifetime. Owing to the lack of movable Br vacancy defects, ion migration was significantly inhibited, and the hysteresis effect of the device was reduced.

### Surface orientation

**Fig. 8:**
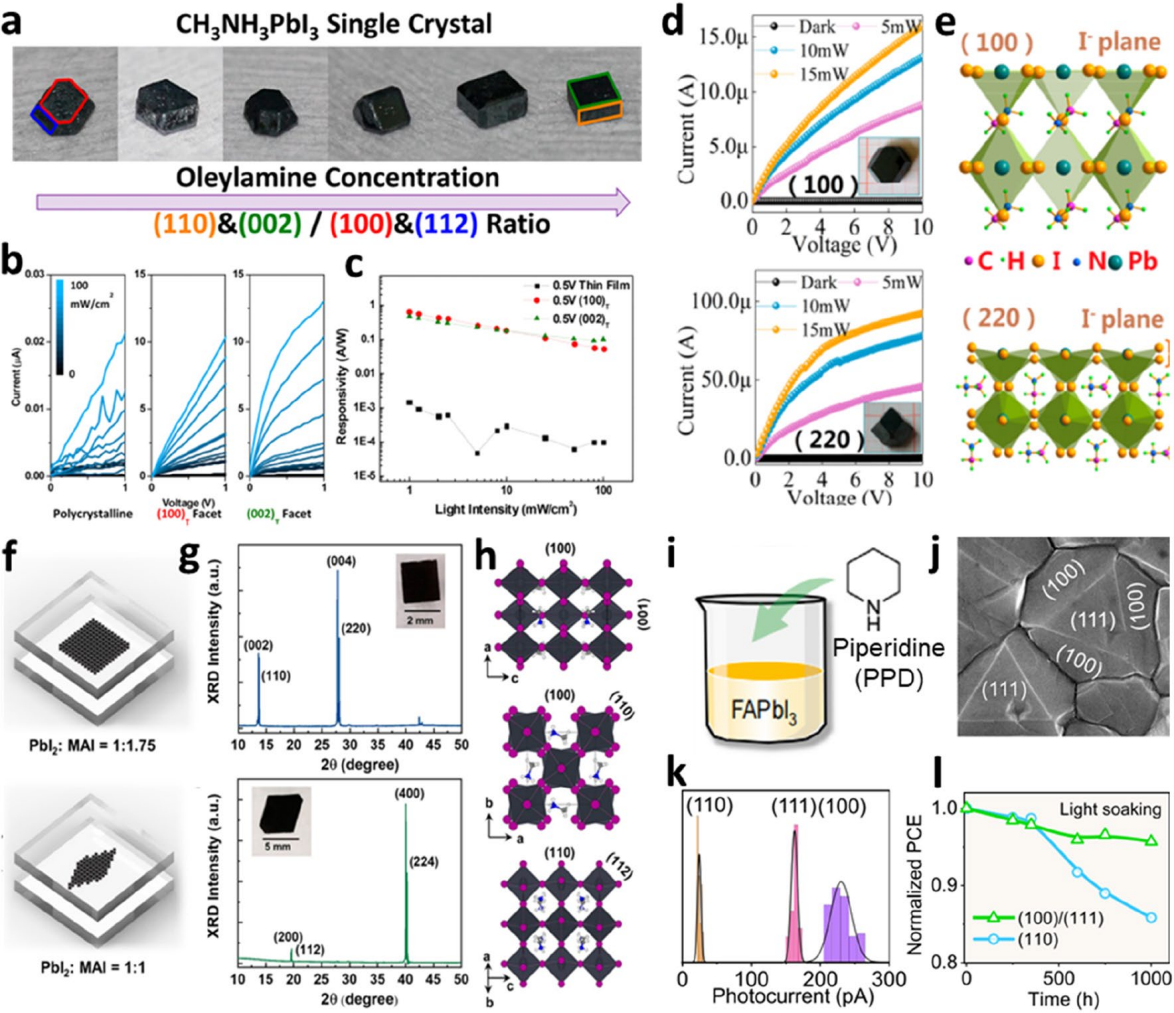
Surface orientation engineering of PVK-SC. **a** Photographs of the MAPbI_3_ SC showing continuous morphological transition triggered by the addition of oleylamine ligand to the solution. **b ***I–V* curves of the MAPbI_3_ SC photodetector. **c** Calculated responsivity of the polycrystalline thin film, SC (100) facet, and SC (002) facet photodetectors. Reproduced with permission from Ref. [77]. Copyright 2017, American Chemical Society. **d** Dark and photocurrents curves of (100) and (220) facet photodetectors, respectively. **e** Diagrams of surface structures on (100) and (220) facets of the MAPbI_3_ SC. [78]. Copyright 2017, American Chemical Society. **f** Schematic of MAPbI_3_ SCTFs with preferred (001) and (100) orientations. **g** XRD patterns of MAPbI_3_ (001) and (100) SCTFs. Insets are photographs of SCTF samples. **h** Diagrams of crystal structures of tetragonal-phase MAPbI_3_ with different exposed facets. Reproduced with permission from Ref. [79]. Copyright 2022, American Chemical Society. **i** Schematic of the α-FAPbI_3_ crystal with three facets. **j** SEM image of the PVK SC film with well-defined facets. **k** Photocurrent for the (100), (111), and (110) facets estimated based on Kelvin probe force microscopy (KPFM) and pc-AFM images. **l** Light stability measurement of the devices with different dominant facets. Reproduced with permission from Ref. [80]. Copyright 2022, Elsevier

An effective way to improve charge transport and electrode extraction in PVK SC photoelectronic devices is to optimize the crystal orientation of the SCTFs to achieve effective carrier extraction [76]. Jao et al. proposed a ligand-assisted crystallization method for preparing PVK SCs with controllable shapes and exposed facets [77]. As shown in Fig. 8a, with increasing concentration of the oleylamine ligand, the PVK SCs underwent a continuous transformation from a dodecahedral to cubic morphology. PVK SC photodetectors with different facet orientations were fabricated. The photocurrent on the (002) facet exceeded that on the (100) facet with increasing voltage because of the faster ion migration (Fig. 8b). The results in Fig. 8c show that the SC photodetectors have higher responsivity than the thin film photodetector. Ding et al. successfully obtained MAPbI_3_ SCs with exposed (220) facets from solution by adjusting the overall supersaturation of the growth solution and reducing the growth rate of the (100) and (112) surfaces [78]. As shown in the *I–V* curve (Fig. 8d), the SC photodetectors with different surface exposure levels exhibited anisotropic optoelectronic properties. A comparison of the surface structure of the (100) and (220) facets (Fig. 8e) showed that the average density of I^−^ ions in the (220) facet was higher than that in the (100) facet. Therefore, a strong photocurrent, high response rate, and high external quantum efficiency (EQE) were observed for the photodetector with (220) facets owing to the I^−^ ion migration involved in the transport. Yang et al. prepared MAPbI_3_ SCTFs with different crystal orientations using the space-confined growth method [79]. As shown in Fig. 8f, different precursor ratios (PbI_2_:MAI) result in different preferred facets. The view of the crystal structures of the different exposed facets in Fig. 8h shows that the molecules are arranged differently in the lattice, leading to anisotropic photoelectric characteristics. The results show that the MAPbI_3_ (001) SCTFs exhibited a reduced PL intensity and a short PL lifetime because of the presence of uncoordinated Pb^2+^ on the surface. After the passivation of the [6]-phenyl-C61-butyric acid methyl ester (PCBM) transport layer, the migration of ions from the PVK to the top surface was inhibited. The MAPbI_3_ (100) facets exhibit improved electron transport characteristics owing to their appropriate interfacial energy arrangement and charge density redistribution. This study emphasizes the importance of optimizing the PVK surface orientation to improve the performance and stability of PVK SCTF devices. Ma et al. synthesized FA-based PVK thin films composed of definite (100) and (111) facets by adding piperidine (PPD) (Fig. 8i) [80]. SEM images of the PVK polyhedral SCs with well-defined facets are shown in Fig. 8j. The carrier mobility and photocurrent of the (100) facets were similar to those of the (111) facet; however, they were significantly higher than those of the (110) facet (Fig. 8k). Therefore, the PVK solar cells with (100) and (111) facets exhibited a high PCE of 24.64%, and the photo-immersion stability remained at 96% of the initial PCE within 1000 h (Fig. 8l). In the latest study, this group further investigated the reason for the water stability of the SC dominated by the (111) facet in the PVK thin films [81]. This provides significant guidance for studying the facet-dependent degradation and facet engineering of highly efficient and stable PVK solar cells.

### Interface modification

**Fig. 9:**
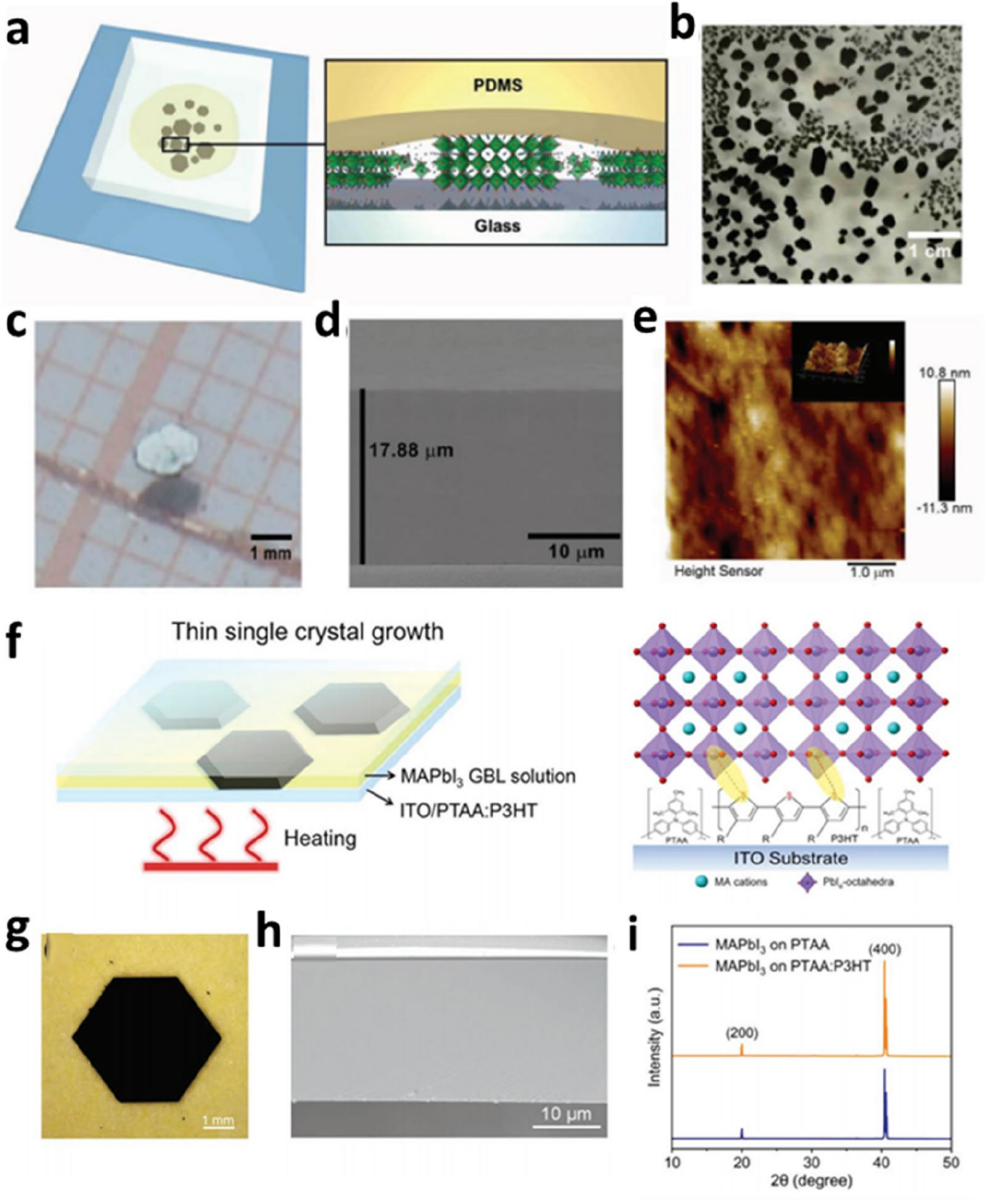
Interface modification engineering of PVK SCTFs. **a** Schematic of (FAPbI_3_)_0.85_(MAPbBr_3_)_0.15_ SCTFs grown via the PDMS-assisted solvent evaporation crystallization method. **b** Photograph of SCTFs covered by PDMS. **c** Microscope image of SCTF. **d** Cross-sectional SEM image of the SCTF. **e** AFM image of the surface of the SCTF. Reproduced with permission from Ref. [83]. Copyright 2018, Wiley-VCH. **f** Schematics of the growth of MAPbI_3_ thin SCs via the space-confined method and interaction between the P3HT molecule and PVK SCTFs. **g** Photograph of a PVK SCTF. **h** Photograph and cross-sectional SEM image of the MAPbI_3_ SCTF. **i** XRD patterns of the MAPbI_3_ SCTFs grown on PTAA (blue line) and PTAA:P3HT (orange line) HTLs. Reproduced with permission from Ref. [84]. Copyright 2021, Wiley-VCH

Another key factor limiting the performance of PVK SCTF devices is the poor interfacial contact between the SC and the surrounding layers. Good contact between the absorbing layer and carrier transport layer leads to effective interface carrier injection. Therefore, optimizing the interface of the SCTF and the electron or hole transport layer is important for improving the performance of PVK SCTF devices [82]. Optimized energy-level matching between functional layers will lead to efficient carrier extraction and can minimize the energy loss. Huang et al. reported a polydimethylsilane (PDMS)-assisted solvent evaporation crystallization method for preparing PVK SCTFs on a NiO_x_/ITO substrate (Fig. 9a) [83]. Owing to its high porosity, the PDMS cover facilitated the slow evaporation of solvents during crystallization. Another advantage of PDMS soft materials is that the removal process does not cause mechanical damage to the surface of the PVK SCTFs. Low roughness leads to a significantly improved quality of the SC surface, which ensures good contact with the transport layer (Fig. 9d,e). Therefore, the PVK SCTF solar cell device had a high PCE. This study focuses on achieving high-quality PVK SC devices through the fine control of the interface. Li et al. added hydrophobic poly(3-hexylthiophene) (P3HT) molecules to the PTAA HTL for preparing PVK SCTFs via an interface modification strategy (Fig. 9f) [84]. The hydrophobic P3HT molecule interacts with undercoordinated Pb^2+^ and promotes ion diffusion during crystallization, resulting in PVK SCTFs with a reduced interface defect density, suppressed nonradiative recombination, and accelerated charge transport. The low *V*_OC_ loss and high short-circuit current density (*J*_SC_) and FF resulted in an increased PCE of the MAPbI_3_ SCTF solar cells. This study proves the importance of interface modification for achieving high-performance PVK SCTFs devices.

## Applications of PVK SCTFs

Owing to the advancement of preparation methods and surface engineering, PVK SCTFs with controllable thickness and low surface defects have been widely applied in photoelectric devices, such as solar cells, photodetectors, lasers, and light-emitting diodes (LEDs) [85–88]. However, the performance of several devices needs to be significantly improved to match or surpass that of polycrystalline devices. Therefore, in-depth studies of the photoelectric mechanism of PVK SCTF devices are crucial.

### PVK SCTF solar cells

The architecture of a solar cell plays a crucial role in determining its performance. To optimize the transfer and collection of photogenerated charges, an appropriate device architecture must be selected [89, 90]. The device architecture of PVK solar cells can be divided into vertical and lateral structures. The vertical structure consists of a transparent electrode, such as ITO and fluorine-doped tin oxide (FTO), an electron transport layer (ETL), an HTL, and a metal electrode. Generally, the HTL must be used as the substrate for the growth of the SC film, and the ETL must be coated or deposited on the surface of the SC film [91]. The inherent advantages of lateral-structured devices are their simple structure, simple manufacture, and low cost [92]. In general, two planar electrodes are deposited on the top surface of an SC on the same side. The separation and transport of charge occur along the plane of the device and are controlled by the electrode geometry. The photons absorbed by the lateral-structured device are close to the surface of the crystal, and the carrier diffusion length of the photogenerated carrier must be sufficiently long to decrease carrier recombination.

**Fig. 10:**
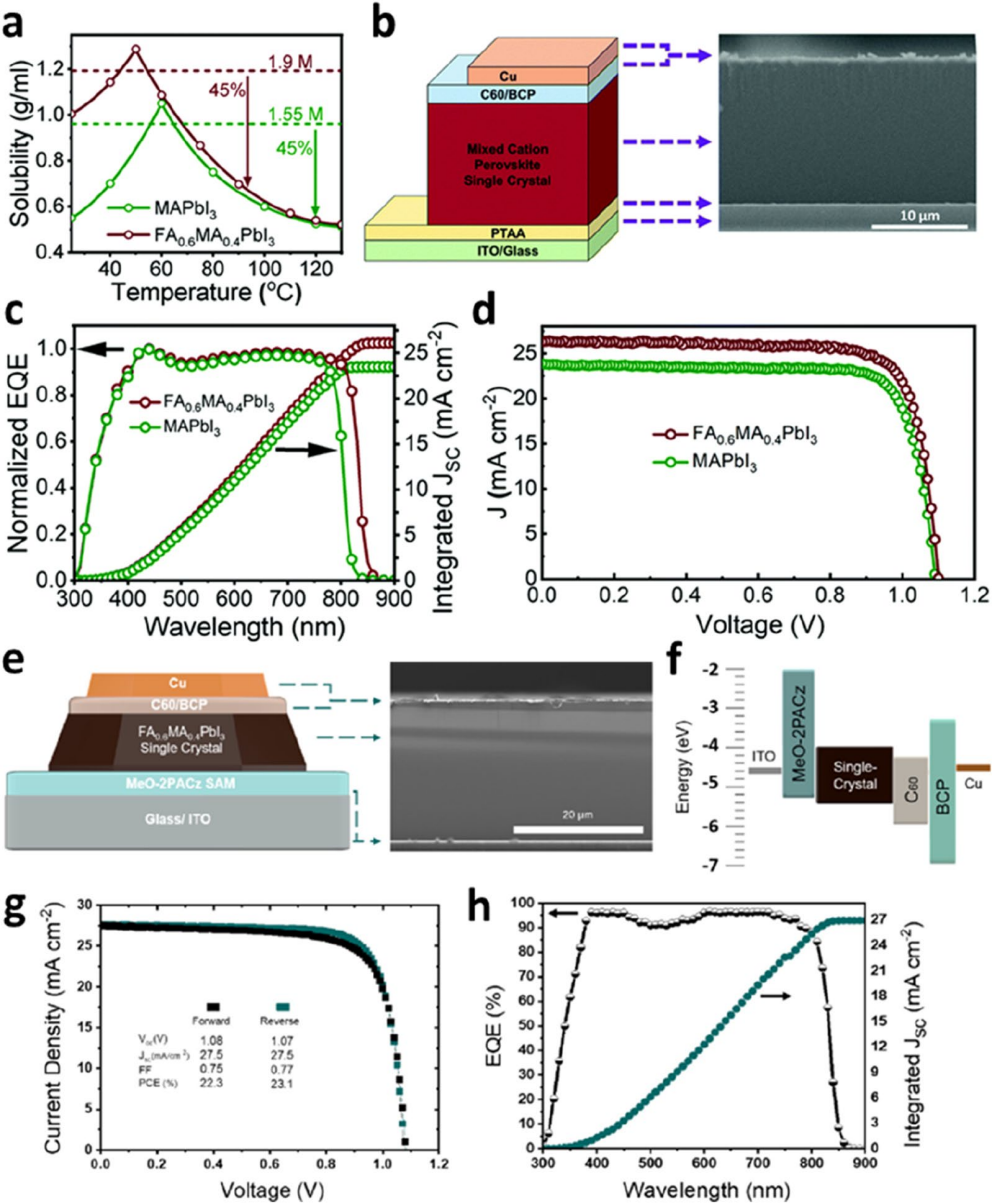
PVK SCTFs for photovoltaic devices. **a** Solubility of FA_0.6_MA_0.4_PbI_3_ and MAPbI_3_ in a GBL solution at different temperatures. **b** Device architecture (left) and cross-sectional SEM image (right) of the mixed cation PVK SC solar cell. **c, d** EQE spectra and comparison of *J–V* curves of MAPbI_3_ and FA_0.6_MA_0.4_PbI_3_ SC devices. All measurements were conducted under 1 sun illumination. Reproduced with permission from Ref. [96]. Copyright 2021, The Royal Society of Chemistry. **e** Device architecture and cross-sectional SEM image of the FA_0.6_MA_0.4_PbI_3_ SC device with a hydrophilic HTL. **f** Diagram of the energy levels of different layers. **g ***J–V* curves for the champion device under 1 sun illumination. **h** EQE spectrum with the integrated *J*_SC_ of the champion device. Reproduced with permission from Ref. [97]. Copyright 2023, American Chemical Society

In 2016, Peng et al. used a cavitation-triggered asymmetric crystallization (CTAC) strategy to grow PVK SCTFs, which were used to fabricate simple photovoltaic devices [93]. Effective photogenerated carrier collection was realized by controlling the thickness of the SCTF within the range of the carrier diffusion length. The internal quantum efficiency (IQE) of the SCTF device without any transport layer was approximately 100%, with a stable PCE of more than 5%. After introducing a TiO_2_ layer as an ETL, the PCE of the SCTF device was enhanced to ~ 6.53%. The TiO_2_ interlayer minimized the traps on the lower surface of the PVK, resulting in less hysteresis. This study broadened the application prospects of PVK SCTFs solar cells and laid a foundation for subsequent research. Rao et al. prepared laminar MAPbBr_3_ SCTFs with high crystallinity, high mobility (23.7 cm^2^ s^− 1^ V^− 1^), and a low defect density (2.5 × 10^10^ cm^− 3^) [94]. Three SCTF solar cells with different structures were fabricated to study the carrier dynamics at the interfaces. The results of transient optical and electrical tests indicated that the FTO/TiO_2_/PVK/HTL/Au device exhibited fast carrier separation and charge extraction, which enhanced the PCE of the PVK SCTF solar cells to 7.11% and improved the long-time operation stability. This study demonstrates the importance of carrier transport layers for efficient PVK SCTF solar cells. In 2017, Chen et al. directly grew MAPbI_3_ SCTFs on two hydrophobic PTAA HTLs, which facilitated growth in the lateral dimension and enhanced the crystallinity [49]. The changes in the absorption and device efficiency related to the thickness of the SCTFs were probed by simulation. The below-bandgap spectral response of the MAPbI_3_ SCTFs was enhanced, leading to an increase in *J*_SC_. This also directly led to an increase in the theoretical maximum of the photocurrent. However, charge recombination dominates the reduction of *V*_OC_ with increasing crystal thickness. Therefore, determining the optimal SC film thickness is critical for obtaining the highest device efficiency. In 2019, Chen et al. increased the PCE of MAPbI_3_ SCTFs solar cells [95]. This device utilized the ITO/PTAA/MAPbI_3_/C60/BCP/Cu structure, achieving a PCE of 21.09% and FF of 84.3%. In 2021, Alsalloum et al. proposed a promising strategy for optimizing the composition of PVK precursors to increase the efficiency of PVK SCTF solar cells (Fig. 10a) [96]. The architecture of this PVK SCTF solar cell is shown in Fig. 10b. The absorption band edge of the SC thin film prepared with mixed cationic FA_0.6_MA_0.4_ was red-shifted by approximately 50 nm (Fig. 10c). The introduction of FA ions reduces the crystallization temperature and limits the escape of MAI, which has been shown to generate a high *V*_OC_ without passivation treatment. Additionally, mixed cationic PVK SCTFs exhibited thermal stability and few defects. Mixed cationic PVK SCTF solar cells exhibited a PCE of 22.8% and *J*_SC_ of over 26 mA cm^− 2^(Fig. 10d). The hydrophobicity of the commonly used PTAA HTL accelerated ion diffusion and promoted crystal growth. However, the carrier mobility was low, and the density of defect states at the buried interface was high. The PVK SCTF was easily separated from the substrate. In the latest study, Bakr et al. achieved PVK SC solar cells with a record-high efficiency of 23.1% and ultrahigh stability using a hydrophilic self-assembled monomolecular layer, namely, MeO-2PACz, as the HTL (Fig. 10e) [97]. The key to aspect of the experiment is the formation of a hydrogen bond between the H atom of the MeO group and the surficial I atom, which interacts with the I_2_-rich PVK SC surface. Compared with PTAA, which has a larger space-blocked molecular structure, MeO-2PACz binds to the PVK surface more strongly, improving the adhesion of SCTF to the substrates. Fig. 10f shows the energy levels of different layers. The current density−voltage (*J*−*V*) characteristics in Fig. 10g and EQE spectrum in Fig. 10h proved that the PVK SCTF solar cells grown on the hydrophilic self-assembled monomolecular layer had high PCE. Their study provided an innovative perspective for the selection of transport layers in PVK SCTFs solar cells. A summary of the reported PVK SCTFs solar cells is presented in Table 1.

**Table 1:** PVK SCTFs solar cells and their performance

Device architecture	Prepared method	Thickness	*J*_SC_ (mA cm^− 2^)	*V*_OC_ (V)	FF (%)	PCE (%)	Year	Refs.
FTO/TiO_2_/MAPbBr_3_/Au	Cavitation-triggered asymmetrical crystallization method	1 μm	6.69	1.36	69.0	6.53	2016	[93]
ITO/PTAA/MAPbI_3_/PCBM/C_60_/BCP/Cu	Space-confined method	10 μm	21.0	1.08	78.60	17.80	2017	[49]
Au/MAPbI_3_/PCBM/Ag	Space-confined method	200 nm	18.33	0.80	32.90	4.83	2017	[54]
FTO/TiO_2_/MAPbBr_3_/Spiro-MeOTAD/Au	Space-confined method	16 μm	8.77	1.31	62	7.11	2017	[94]
FTO/TiO_2_/MAPbI_3_/Spiro-OMeTAD/Ag	Space-confined method	∼3 μm	22.28	0.68	59	8.78	2017	[91]
ITO/NiO_x_/(FAPbI_3_)_0.85_(MAPbBr_3_)_0.15_/TiO_2_/Ag	Space-confined method	24.5 μm	23.14	1.03	51	12.18	2018	[83]
ITO/PEDOT:PSS/MAPbI_3_/PCBM/Ag	Space-confined method	50 μm	22.15	0.75	27	4.40	2018	[98]
ITO/PTAA/MAPbI_3_/C_60_/BCP/Cu	Space-confined method	20 μm	23.46	1.076	83.5	21.09	2019	[95]
Au/MAPbI_3_/C_60_/BCP/Au	Surface tension-assisted method	38 μm	5.06	0.66	44	5.90	2019	[57]
ITO/PTAA/MAPbI_3_/C_60_/BCP/Cu	Low-temperature space-confined method	∼20 μm	23.68	1.144	81	21.93	2020	[72]
ITO/PEDOT:PSS/MAPbI_3_/PCBM/BCP/Ag	Antisolvent vapor-assisted crystallization method	300 nm	22.60	1.08	82.5	20.1	2020	[51]
PET/ITO/SnO_2_/MAPb_0.5+x_Sn_0.5x_I_3_/Spiro-OMeTAD/ Au/PDMS/Su 8	Lithography-assisted Epitaxial-growth method	2 μm	24.0	1.05	79.5	20.04	2020	[87]
Au/MAPbI_3_/C_60_/BCP/Au	Space-confined method	–	22.49	0.93	55.1	11.52	2020	[74]
ITO/PTAA/FA_0.6_MA_0.4_PbI_3_/C_60_/BCP/Cu	Space-confined method	15 μm	26.2	1.10	79	22.80	2021	[96]
ITO/PTAA:P3HT/MAPbI_3_/C_60_/BCP/Cu	Space-confined method	20 μm	23.88	1.13	81.8	22.1	2022	[84]
ITO/PTAA/FA_0.55_MA_0.45_PbI_3_/C_60_/BCP/Cu	Space-confined method	20 μm	22.75	1.062	76.2	18.41	2022	[99]
ITO/PTAA/MAPbI_3_(100)/PCBM/C_60_/BCP/Cu	Space-confined method	20–25 μm	23.8	1.04	78	19.3	2022	[79]
ITO/PTAA/MAPbI_3_(001)/PCBM/C_60_/BCP/Cu	Space-confined method	20–25 μm	20.1	0.98	81.4	16.0	2022	[79]
ITO/MeO-2PACz/ FA_0.6_MA_0.4_PbI_3_/PCBM/C_60_/BCP/Cu	Space-confined method	∼20 μm	27.5	1.07	77	23.1	2023	[97]
ITO/PTAA/MAPbI_3_/3-mercaptopropyl(dimethoxy)methylsilane (MDMS)/C_60_/BCP/Cu	Space-confined method	40 μm	24.1	1.13	81.5	22.2	2023	[22]

### PVK SCTF photodetectors

**Fig. 11:**
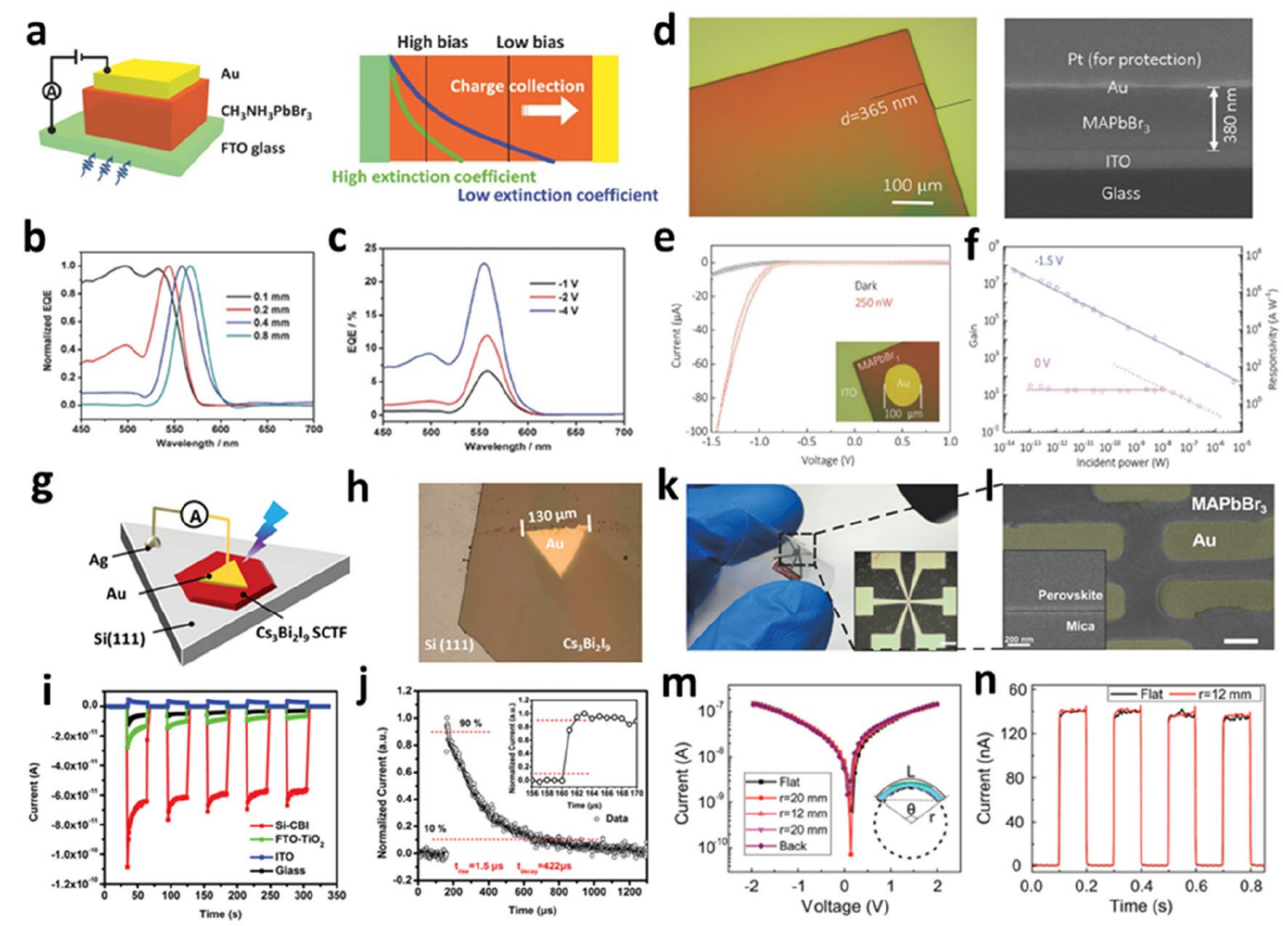
PVK SCTFs for photodetector devices. **a** Schematic of the device architecture (left) and the mechanism of operation of a narrow-band photodetector (right). **b** Normalized EQE spectra of MAPbBr_3_ SCTF photodetectors with different thicknesses under a bias of − 1 V. **c** EQE spectra of 0.4 mm-thick device under various biases. Reproduced with permission from Ref. [101]. Copyright 2017, Wiley-VCH. **d** Optical image (left) and cross-sectional SEM image (right) of the MAPbBr_3_ SCTF. **e ***I–V* curves of a photodetector under dark and 250 nW illumination conditions. **f** Gain and responsivity of the MAPbBr_3_ SCTF photodetector. Reproduced with permission from Ref. [50]. Copyright 2018, Wiley-VCH. **g** Schematic of the device architecture of the Si(111)–Cs_3_Bi_2_I_9_ SCTF photodetector. **h** Optical image of the device. **i** Comparison of self-powered performances of different photodetectors. **j** Rise and decay times estimated using the photoresponse to 355 nm laser pulse at 3 V bias. Inset shows the enlarged curve. Reproduced with permission from Ref. [103]. Copyright 2021, Wiley-VCH. **k** Photograph of the flexible photodetector. Inset shows a micrograph of the device (scale bar is 100 μm). **l** False-color SEM image of the device (scale bar is 3 μm). **m ***I−V* curves of the device as a function of the bending radius. **n ***I−t* curves of the device exposed to pulsed light illumination before and after bending at a bias of 2 V. Reproduced with permission from Ref. [106]. Copyright 2020, American Chemical Society

A photodetector is a device that converts an optical signal into an electrical signal through the photoelectric effect to detect the incident light of different intensity and wavelength. Under an externally applied electric field, electrons and holes are separated, and charge carriers are transferred to the electrode. Photodetectors are widely used in scientific research and industrial applications, such as medical imaging, optical communication, biosensing, and environmental monitors. However, high-performance photodetectors with specific functions are expensive and poorly integrated. PVK materials have excellent photoelectric properties and are prepared via low-cost procedures, providing new methods for the fabrication and development of new, integrated, and high-performance photodetectors [100]. Huang et al. reduced the thickness of PVK SCs and obtained a vertical-structured p-i-n photodetector based on PVK SCTFs [101]. SC films grown in situ on the transport layer had a lower defect density and longer carrier lifetime than polycrystalline films. The reduction in the grain boundary numbers in SCTFs resulted in less carrier recombination. The PVK SCTF photodetector had the characteristics of a low dark current, low noise equivalent power, and high specific detection rate, which prove the significant application potential of the SCTF in photodetectors. Kuang’s group prepared large-area SC films using an efficient space-confined crystallization method to fabricate high-performance narrow-band photodetectors (Fig. 11a) [102]. It was demonstrated that the narrow-band response was closely related to the light penetration length and charge diffusion length over the entire spectral range. As shown in Fig. 11b and c, increasing the applied bias or decreasing the thickness can increase the carrier drift length and consequently enhance the efficiency of charge collection on the electrode, thereby improving the performance of the photodetector. This filter-free narrowband PVK photodetector is expected to play a key role in imaging, machine vision, optical communication, and other fields. Yang et al. significantly improved the crystal quality and reduced the thickness of SC thin films by optimizing the preparation conditions for confined growth [50]. The thickness of the SC film reached several hundred nanometers (Fig. 11d). The MAPbBr_3_ SCTF photodetector achieved a high photoconductivity gain and photosensitivity. The results showed that PVK SCTFs have broad development prospects in the field of optoelectronic devices.

Li et al. elucidated the key role of supersaturation in the growth kinetics of lead-free halide PVK Cs_3_Bi_2_I_9_ SCTFs via *in-situ* observation [103]. Controlling the supersaturation of the solution can reduce the nucleation density of inverse-temperature crystallization and prolong the growth of SC films. A high-performance photodetector was prepared by combining a Cs_3_Bi_2_I_9_ SCTF with a Si substrate (Fig. 11g). Finally, good lattice matching and band alignment between the Si (111) and Cs_3_Bi_2_I_9_ (001) surfaces facilitated photogenerated charge ionization and extraction, resulting in a significant increase in the photosensitivity compared to that of photodetectors based on other substrates. Yan et al. introduced a simple solution epitaxial growth method for preparing MAPbBr_3_ SCTFs with controllable thickness and high quality on MAPbCl_3_ SCs [104]. Notably, a p-n heterojunction was formed on the heteroepitaxial interface of the SC film, and transverse heterojunction photodetectors were prepared by depositing Au as an electrode. Owing to the internal electric field of the heterojunction, heterojunction photodetectors exhibit clear rectification behavior in imaging and X-ray detection without external power. They also exhibit a high response rate, short response time, and high sensitivity. PVK SCTFs can be used to produce heterogeneous photodetectors with excellent properties. Xu et al. used a polymer dry transfer technique to transfer SCTFs to MoS_2_ to construct a vertical heterostructure and obtain a high-performance, self-powered photodetector.

Liu et al. also investigated inch-level, flexible PVK SCTF photodetectors [105]. Two-dimensional PVK SCTFs with an area of more than 2500 mm^2^ and a thickness as low as 0.6 μm were prepared via induced peripheral crystallization. This ultrathin flexible SCTF has an ultralow defect density, high uniformity, and long-term stability. This provides a new approach for developing PVK SCTFs for application in the field of flexible electronic devices. Jing et al. used mica sheets with atomically smooth surfaces and excellent wettability as growth substrates [106]. PVK SCTF with a thickness of 20 nm was prepared using two newly cracked mica sheets to form a limited growth space. A photograph of the flexible device is shown in Fig. 11k. By reducing the thickness of the SCTF, the response rate of the flexible photodetector was significantly improved. This solution-based synthesis process renders it possible to manufacture flexible photodetectors at room temperature. Polarized light detection is important in optical communication, optical switches, polarization sensors, and optical radars. Zhang et al. used a nondestructive solution epitaxial growth method to prepare PVK SCTFs with structured surfaces [107]. These SCTFs exhibited photoelectric properties related to the anisotropy caused by the patterned structures. The polarization-sensitive photodetector prepared via this method showed a high polarized-light detection sensitivity of ~ 2.2 and a high light detection response and external quantum efficiency under linearly polarized light at 532 nm. Li et al. developed a moiré PVK polarized photodetector with *in-situ* encapsulation [108]. The advantage of this photodetector is that the SC surface has a double nanograting structure, which improves the absorption of the incident light, resulting in the improved responsiveness and polarization sensitivity of the device. The PDMS encapsulation enhanced the water stability and flexibility. In addition, low-cost, solution-treated, high-atomic-number PVK SCs radiation detectors have extraordinary potential in the fields of biomedical sensing and imaging, nondestructive testing of industrial products, and safety inspection [104, 109, 110]. Many PVK SCs radiation detectors with high sensitivity, high resolution, and low radiation dose have been successfully prepared. However, the effective radiation can not be efficiently absorbed by PVK SCTFs with thin thickness. Therefore, to promote the development of PVK SCTFs radiation detector, it is necessary to optimize the equipment structure, suppress the surface recombination caused by surface defects, and improve the operational stability.

### PVK SCTF light-emitting devices

**Fig. 12:**
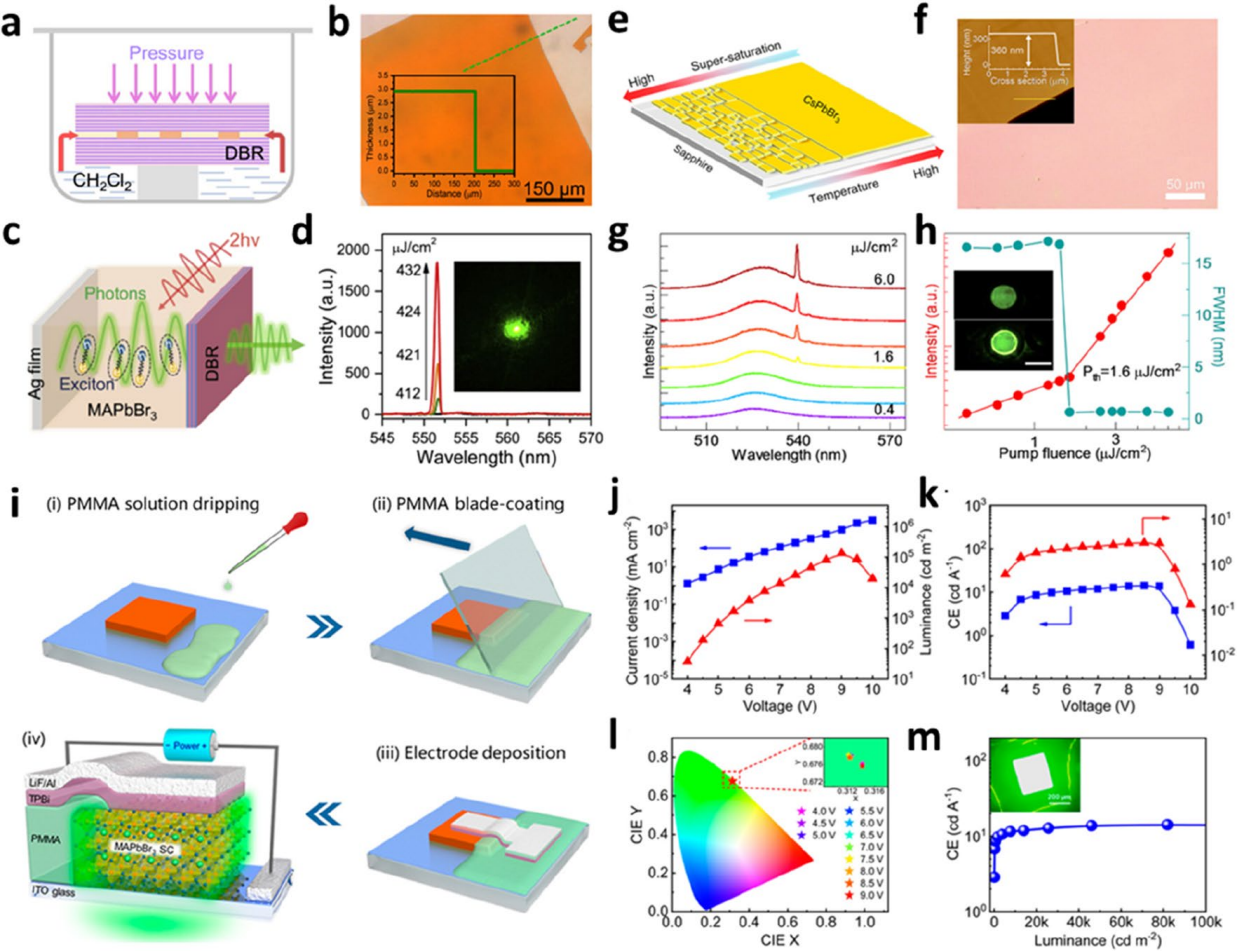
PVK SCTFs for light-emitting devices. **a** Schematic of the growth of MAPbBr_3_ SCTFs. **b** Optical image and height profile of the MAPbBr_3_ SCTF. **c** Diagram of exciton–photon interaction in the DBR laser. **d** PL spectrum of the microcavity structure measured as a function of the pump fluence. Inset shows the dark-field image of the lasing emission. Reproduced with permission from Ref. [115]. Copyright 2019, Elsevier. **e** Schematic of the nucleation process of CsPbBr_3_ SCTFs on a c-plane sapphire substrate. **f** Optical images of CsPbBr_3_ SCTFs. Inset shows the AFM image and height profile of the CsPbBr_3_ SCTF. **g** Power-dependent PL of the CsPbBr_3_ microdisk with increasing pump fluence (from 0.4 to 6 µJ cm^− 2^). **h** Pump-fluence-dependent PL intensity and FWHM of the CsPbBr_3_ microdisk. Inset shows the PL image of the microdisk below and above the threshold (scale bar is 15 μm). Reproduced with permission from Ref. [116]. Copyright 2020, American Chemical Society. **i** Schematic of the LIB method and device architecture of LED. **j** *L–V–J* curve of the brightest device. **k** CE and EQE data of the brightest device as a function of voltage. **l** CIE diagram of the brightest device. **m** CE versus the luminance curve of the device. Inset shows the EL image of the device. Reproduced with permission from Ref. [119]. Copyright 2022, American Chemical Society

PVK SC materials have a high refractive index, high optical gain, tunable wavelength, and high PLQY, rendering them promising for fabricating light-emitting devices [111–114]. Li et al. reported an inverse solvent-assisted space-confined growth method (Fig. 12a) [115]. A single-mode vertical-cavity surface-emitting laser (VCSEL) was fabricated by integrating a large-area and high-quality MAPbBr_3_ SCTF with a distributed Bragg mirror (DBR) and a Ag mirror (Fig. 12c). Under the action of two-photon pumping, a strong coupling of exciton-polarization excitons was observed, and an intense laser was emitted with the enhancement of pumping. This VCSEL exhibits a low threshold, high quality (Q) factor, and small divergence angle. PVK SCTFs have broad development prospects in the fields of coherent light sources and multifunctional integrated photoelectric devices. Zhong et al. demonstrated a large-area CsPbBr_3_ SC film prepared on a sapphire substrate (Fig. 12e) [116]. The SCTF exhibited well-amplified spontaneous radiation properties. A microdisk array was fabricated via focused ion-beam etching, and a single-mode laser was constructed (Fig. 12g). Similarly, Liu et al. prepared PVK SCTFs with controllable thickness via the confined solution growth method [117]. A distributed feedback (DFB) laser was fabricated using the focused ion beam method based on surface-patterned SCTFs. The performance and stability of this type of laser are higher than those of polycrystalline thin films because SCTFs are resistant to ion beam etching. PVK SCs have the advantages of a long carrier diffusion length, no grain boundary scattering, and atomic-level surface flatness, which are conducive to realizing room-temperature continuous lasers and electrically pumped lasers.

Lai et al. reported a PVK micro-SC LED with a simple metal–insulator–semiconductor structure [118]. The purpose of introducing an insulating layer is to fill the gaps between the crystals to avoid leakage. This type of LED based on PVK microcrystals showed high brightness. However, after a long operation time, the device broke down, and no radiation from the excited states was observed. In situ microscopic observation showed that the device temperature increased, and the material was degraded at a high current density. The severe Joule heating effect under large injection conditions is the main issue to be resolved in the realization of an electronically pumped PVK microcrystal laser. Zhang et al. reported a simple process for preparing a liquid-insulated bridge (LIB) for high-performance PVK SCTF LEDs (Fig. 12i), achieving ultrahigh brightness and a long half-life [119]. In detail, the preparation involves covering one edge of the SC film with a polymethyl methacrylate (PMMA) solution via the scraping coating method and then depositing the injected layer and electrode material. The reliable interlayer contact caused by the PMMA insulation aids the mitigation of the difficulties in constructing PVK-based microcrystal LEDs and significantly improves the device performance. This low-cost and simple process avoids the damage to PVK materials caused by organic solvents and ultraviolet light during the photolithography process, emphasizing the application potential of PVK materials in the field of luminescence. In order to solve the limitation of ion migration and Auger recombination on the performance of PVK SCTFs LEDs, Xiao et al. fabricated MA_0.8_FA_0.2_PbBr_3_ SCTFs LEDs with smooth surface, high crystallinity, and low trap density using mixed cations and adding excess organoammonium halides and polyvidone to the precursor [120]. PVK SCTFs LEDs with a thickness of 1.5 μm exhibit a high luminance of 86,000 cd m^− 2^ and a peak external quantum efciency of 11.2%. The extrapolated *T*_50_ lifetime for PVK SCTFs LEDs reached a value of 12,500 h at an initial luminance of 100 cd m^− 2^ owing to suppressed ion migration. This research will prove useful in expanding our understanding of how to increase the lifetime of PVK SCTFs LEDs for practical applications. A summary of the reported PVK SCTFs LEDs is presented in Table 2.

**Table 2:** PVK SCTFs LEDs and their performance

Device architecture	Prepared method	Thickness	EL (nm)	Peak EQE (%)	L_max_ (cd m^− 2^ )	*T*_50_ Stability	Refs.
ITO/MAPbBr_3_/TPBi/LiF/Al	Space-confined method	1 μm	548 nm	11.2	86,000	12,500 h at an initial luminance of 100 cd m^− 2^. (Extrapolated)	[119]
ITO/Poly(*N*-vinylcarbazole)/MAPbBr_3_/TPBi/LiF/Al	Space-confined method	1.5 μm	548 nm	3.0	136,100	88.2 min at an initial luminance of ~ 1100 cd m^− 2^ .	[120]

### PVK SCTF artificial synapse and field-effect transistor

PVK SCTFs have also been used to prepare other optoelectronic devices. Tian et al. reported resistance storage based on 2D Rudderstein–Popper phase mixed lead bromide 2D PVK SC materials [121]. Ultrathin 2D PVK materials with good ion migration and diffusion were obtained via a simple physical stripping method. As a type of artificial synaptic device, this 2D PVK SCTF exhibits a substantially low operating current and power consumption, reaching the level of a biological synapse. This also demonstrates the application potential of the PVK SCTF in the field of neuromorphic circuits and systems. The mechanical method leads to an uncontrolled thickness of the thin film and probably breaks the materials. Gong et al. introduced a seed crystal-induced domain-limited growth method for preparing an MAPbBr_3_ SCTF with a controlled thickness and transverse size, low surface roughness, low defect state density, and high crystal quality [122]. Subsequently, transverse-structure artificial synapses were fabricated based on the SCTF to achieve a series of synaptic functions aimed at controlling the channel distance and reducing the working current and energy consumption. Their study provided new insights into the development of artificial synapses in neuromorphic bioelectronics. Yu et al. demonstrated a spatially constrained inverse-temperature crystallization strategy for the synthesis of micron-thin PVK SCs [123]. The PVK SCTF was integrated into bipolar transistors, affording field-effect mobilities of 4.7 and 1.5 cm^2^ V^− 1^ s^− 1^ in p- and n-channel devices, respectively, at room temperature. This type of transistor also had high switching ratios of 10^4^–10^5^ and a low opening voltage. Their study further extended the applications of PVK SCTFs.

## Summary and perspectives

PVK SCTFs with optimized thickness, no grain boundaries, and high crystallinity show significant application potential in the fields of solar cells, photodetectors, light-emitting devices, and other photoelectronic devices. However, the future development of PVK SCTFs is limited by several challenges. First, more research is needed to explore the growth of multicomponent PVK SCTFs. Mixed cationic PVK components can effectively extend the light absorption range of PVK [124–126]. Doping appropriate metal ions into PVKs to release the lattice strain or replace defect sites is an effective method to reduce defects and improve the stability of PVK SCTFs. However, differences in component solubility lead to different crystallization thermodynamics and kinetics, resulting in the formation of PVK or non-PVK phases in the solution. The synthesis of PVK SCs with high quality and an adjustable composition is challenging owing to phase separation in complex solution systems. Second, manufacturing ultrathin and large-area PVK SCTFs for scalable and commercial production remains an unresolved issue [127]. Thus far, no industrial equipment has been shown to fabricate an SCTF with a high aspect ratio by effectively and simultaneously controlling the lateral-dimension size and thickness. Additionally, the production of high-quality and large-area PVK SCTFs on different substrates and transport layers remains challenging, which limits the possibility of integrating PVK SCTF devices with other semiconductor devices [128]. Furthermore, the application of PVK SCTFs in integrated circuits is a significant challenge. Therefore, PVK SCTFs should receive the same degree of attention as polycrystalline PVK films. Considering the superior photoelectric performance and stability of PVK SCs compared to their polycrystalline counterparts, the progress in research on SCTFs devices is expected to exceed that of polycrystalline film devices. A low-cost and large-area manufacturing strategy is essential for realizing the practical application of PVK SC photoelectric devices. Third, PVK SCTFs have significantly more surface defects than polycrystalline films, which leads to carrier recombination on the crystal surface in the former. An energy level mismatch is also observed at the surface interface between the transport layer and PVK SC. Furthermore, the direct contact between the metal electrode and the PVK SC would result in a short circuit. The properties of PVK thin-film devices are also affected by the anisotropy of the crystal structure, crystal orientation, and the degree of lattice matching with the growth surface [129–131]. Poor lattice matching leads to higher stress during crystal growth, resulting in more surface and bulk defects. The development of optimized surface engineering methods will significantly promote the use of efficient and stable PVK SCTF photoelectric devices as excellent alternatives to semiconductor devices in future.

## Data Availability

Not applicable.
